# Erectile dysfunction and lower urinary tract symptoms: a consensus on the importance of co-diagnosis

**DOI:** 10.1111/ijcp.12176

**Published:** 2013-04-25

**Authors:** M Kirby, C Chapple, G Jackson, I Eardley, D Edwards, G Hackett, D Ralph, J Rees, M Speakman, J Spinks, K Wylie

**Affiliations:** 1 Faculty of Health and Human Sciences, University of HertfordshireHatfield, UK; 2 The Prostate Centre32 Wimpole Street, London, UK; 3 Department of Urology, Sheffield Teaching Hospitals NHS Foundation Trust, Sheffield Hallam UniversitySheffield, UK; 4 Department of Cardiology, Guys and St Thomas Hospitals LondonLondon, UK; 5 St James University HospitalLeeds, UK; 6 White House SurgeryChipping Norton, Oxon, UK; 7 Good Hope HospitalBirmingham, UK; 8 Institute of Urology, St Peter's Andrology CentreLondon, UK; 9 Backwell and Nailsea Medical GroupNorth Somerset, UK; 10 Department of Urology, Taunton and Somerset NHS Foundation TrustTaunton, UK; 11 Court View Surgery2a Darnley Road, Strood, UK; 12 Department of Urology, Royal Hallamshire HospitalSheffield, UK

## Abstract

Despite differences in design, many large epidemiological studies using well-powered multivariate analyses consistently provide overwhelming evidence of a link between erectile dysfunction (ED) and lower urinary tract symptoms (LUTS). Preclinical evidence suggests that several common pathophysiological mechanisms are involved in the development of both ED and LUTS. We recommend that patients seeking consultation for one condition should always be screened for the other condition. We propose that co-diagnosis would ensure that patient management accounts for all possible co-morbid and associated conditions. Medical, socio-demographic and lifestyle risk factors can help to inform diagnoses and should be taken into consideration during the initial consultation. Awareness of risk factors may alert physicians to patients at risk of ED or LUTS and so allow them to manage patients accordingly; early diagnosis of ED in patients with LUTS, for example, could help reduce the risk of subsequent cardiovascular disease. Prescribing physicians should be aware of the sexual adverse effects of many treatments currently recommended for LUTS; sexual function should be evaluated prior to commencement of treatment, and monitored throughout treatment to ensure that the choice of drug is appropriate.

**What's known**

Erectile dysfunction (ED) and lower urinary tract symptoms (LUTS) occur frequently as men age. Both conditions have common pathogenetic mechanisms and epidemiological data support a link between ED and LUTS in men that is independent of age and other co-morbidities.

**What's new**

For all men presenting with either ED or LUTS, co-diagnosis of the other condition should be considered. Men should be asked about their sexual or urinary health, as appropriate, and other possible co-morbid conditions should also be investigated. Recognition of the link between ED and LUTS, and further links with other co-morbid conditions, will improve patient and partner quality of life as well as reducing patient morbidity and mortality.

## Introduction

Erectile dysfunction (ED), defined as the inability to maintain and achieve an erection for satisfactory intercourse (1), has a high prevalence and incidence worldwide. A systematic review of epidemiological evidence undertaken in 2002 (2) showed a clear linear increase in prevalence with advancing age, with rates for men younger than 40 years ranging from approximately 2–9%, compared with 18–86% for those older than 80 years. In the Cologne Male Survey (3), overall prevalence of ED was 19.2% with a steep age-related increase from 2% in the 30–39 years age group to 53% in the 70–80 years age group. In the UrEpik study (4), overall prevalence of ED was 21% with a statistically significant linear increase with age (p < 0.001). Although ED is not life threatening, it may be a precursor of more serious conditions, particularly coronary artery disease (CAD). Inman et al. (5) have shown that when ED occurs in younger men, it is associated with a marked increase in the risk of future cardiac events and that overall ED may be associated with an approximately 80% higher risk of subsequent CAD.

Lower urinary tract symptoms (LUTS) in men are caused by a group of disorders affecting the prostate and bladder that share a similar clinical manifestation. National Institute of Health and Clinical Excellence (NICE) guidelines define LUTS as comprising storage, voiding and postmicturition symptoms affecting the lower urinary tract (6). Voiding symptoms include weak or intermittent urinary stream, straining, hesitancy, terminal dribbling and incomplete emptying. Storage symptoms include urgency, increased frequency, urgency incontinence and nocturia. The major postmicturition symptom is postmicturition dribbling, which is common and bothersome. Although LUTS do not usually cause severe illness, they can considerably reduce men's quality of life, and may point to serious pathology of the urogenital tract (6). Storage LUTS are often more prevalent and more bothersome than voiding LUTS (7,8) and are often related to underlying bladder dysfunction that may be secondary to benign prostatic enlargement (BPE), or may arise because of other factors affecting bladder physiology (9). Clinicians should consider all possible causes of LUTS prior to treatment: These include problems with fluid intake, medical conditions such as diabetes or heart failure, and other urological conditions such as overactive bladder or urethral stricture. However, the most common association of male LUTS is BPE secondary to benign prostatic hyperplasia (BPH)^1^ (10). The prevalence of BPH increases with age and approximately 25–50% of men with BPH have LUTS (6). In a large retrospective cohort study of 80,774 males who contributed 141,035 person-years of follow-up, the overall incidence rate of LUTS/BPH was 15 per 1000 man-years (95% CI: 14.8–16.1). The incidence increased linearly with age to a maximum of 38 patients per 1000 at the age of 75–79 years (95% CI: 34.1–42.9) (11). Other studies have shown that LUTS can occur in 15–60% of men older than 40 years of age (12–15), and bothersome LUTS can occur in up to 30% of men older than 65 years (6). Although figures differ from study to study as a result of different definitions and data collected, LUTS are experienced by a large number of men potentially requiring treatment; this figure will continue to rise with increasing life expectancy and the resulting growth of the elderly population (6).

Preservation of sexual function is an important component of quality of life and needs to be considered sympathetically as part of the management of male patients (16). In general, both ED and LUTS affect sexual function and hence quality of life. An international study, designed to examine men's attitudes and behaviours in relation to their ED, emphasised the importance of the couple relationship, and strengthened the view that ED may matter to men because of its significant impact on valued partner relationships (17). This point is illustrated by results from a multinational epidemiological study of female partners of men with ED, which showed a significant decline in desire, arousal, orgasm and satisfaction following the onset of ED (18). Results from the large EpiLUTS study found that sexual enjoyment declined and sexual activity decreased with increasing LUTS; of the 2954 respondents reporting a combination of voiding, storage and postmicturition symptoms at least sometimes, 28.8% said that their sexual enjoyment was ‘somewhat’, ‘quite a bit’ or ‘a great deal’ decreased because of LUTS, and 24.8% had decreased or stopped sexual activity because of LUTS (19). The multinational UrEpik study (4) investigated the impact of LUTS on quality of life and showed that the presence of LUTS in a patient also had an adverse effect on quality of life for the partner. In a later study, results were similar; Mitropoulus et al. (20) studied more than 50 couples in which the male partner suffered from LUTS and showed that all partners suffered with some reduction in quality of life that was directly related to this condition. The most common morbidities were related to the psychological burden of the condition, inadequate sex life, disruption of social life and sleep disturbance. Sells et al. (21) developed a disease-specific questionnaire to assess morbidity in the partners of patients with LUTS and as a result were able to confirm the presence of significant morbidity; almost all partners experienced some morbidity as a consequence of the patients’ conditions, with the most common issues being sleep disturbance, fear of cancer and surgery, social disruption and deterioration in sex life.

There is considerable evidence that LUTS and sexual dysfunction are strongly linked (Table 1). Recognition of these links can be important because:

**Table 1:** Epidemiological evidence from community studies (ranked by sample size)

Reference	Country/ies	Sample *n*	Relevant results for an association between ED and LUTS
McVary et al. (62)	USA	81,659	The baseline prevalence of recorded BPH was 1.5% among men with ED. During the follow-up period (mean 2.2 years), 7.6% had documented BPH
Rosen et al. (30) MSAM-7	USA and six European countries	12,815	Sexual disorders and ‘bothersomeness’ were strongly related to both age and severity of LUTS. When controlling for age, LUTS severity was by far the strongest predictor of ED, with an odds ratio for severe versus mild LUTS of 8.90 (6.85–11.55)
Wein et al. (19) EpiLUTS	USA, UK, Sweden	11,834	26% of men with LUTS had mild to severe ED; men with multiple LUTS had more severe ED and more frequent EJD and PE
Morant et al. (31)	UK	11,217	Compared with men with no LUTS, odds ratios (95% CI) for ED were as follows: storage LUTS 3.0 (2.6–3.4); voiding LUTS 2.6 (2.4–2.7); and both voiding and storage LUTS 4.0 (3.4–4.8). Among the 11,327 men with any recorded LUTS and ED, LUTS diagnosis preceded ED in 63.1% of patients by a mean of 4.8 years
Rosen et al. (63)	USA	6924	In 3084 sexually active men, age, total IPSS, IPSS bother score, hypertension, diabetes and black race/ethnicity were independent predictors of both ED and EJD (all p < 0.05)
Braun et al. (3)	Germany	5000	LUTS was an independent risk factor for ED: LUTS prevalence 72.2% in men with ED, 37.7% in men without ED
Boyle et al. (4) UrEpik	UK, the Netherlands, France and Korea	4800	Diabetes (odds ratio of 1.57, 95% CI: 1.09–2.25) liver problems 1.55 (1.03–2.33); LUTS 1.39 (1.10–1.74); and hypertension 1.38 (1.09–1.75) were significantly correlated with ED
Blanker et al. (64) Krimpen Community Cohort	The Netherlands	3924	ED relative risk of 1.8–7.5 for increasing urinary complaints; risk of ED greater with LUTS than with smoking or cardiac symptoms
Hansen (65)	Denmark	3442	Logistic regression analysis showed LUTS was an independent risk factor for sexual dysfunction in men aged 40–65 years
Ponholzer et al. (66)	Austria	2858	The presence of LUTS was an independent risk factor for the presence of ED; in multivariate analysis controlling for age, co-morbidities and lifestyle, the IPSS (p = 0.0001), the obstructive score of the IPSS (p = 0.0001), nocturia (p = 0.04), and the LUTS bother score (p = 0.002) correlated with the presence of ED (IIEF-5 score < 22)
Rosen et al. (51) BACH survey	USA	2301	ED was significantly associated with LUTS, nocturia and prostatitis in bivariate associations, and with prostatitis in multivariate analyses, controlling for the effects of diabetes and other co-morbidities
Chung et al. (67)	USA	2115	Sexual function domains of LUTS severity questionnaire were inversely associated with the severity of LUTS (all p < 0.001)
Macfarlane et al. (68)	France	2011	Sexual satisfaction had a negative correlation with LUTS: IPS score 0 = relative risk 1, IPSS score > 19 relative risk 3.3
Frankel et al. (69)	UK + 12 others	1694	Sexual dysfunction was strongly associated with LUTS
Blanker et al. (64)	The Netherlands	1688	Multiple logistic regression analyses showed the following correlated with ED: age, smoking, obesity, LUTS, treatment for cardiovascular problems and COPD
Shiri et al. (32)	Finland	1683	Relative risk of LUTS was higher in men with moderate ED (relative risk of 1.5, 95% CI: 1.0–2.3) or severe ED (relative risk of 2.3, 95% CI: 1.4–3.8) than in those free of ED
Song et al. (70)	China	1644	The total IIEF-5 score significantly correlated with the total IPSS score (p < 0.01), the severity of LUTS correlated with the severity of ED (p < 0.01)
Vallancien et al. (71)	Europe	1274	ED was strongly related to age, lower urinary tract symptom severity, body mass index, hypertension and concomitant treatment with calcium channel antagonists; 70% of patients with severe LUTS had ED
Li et al. (72) Asian Survey of Aging Males (ASAM)	Asia	1155	Sexual disorders increased with age and increasing severity of LUTS. Erectile problems were present in 33%, 61% and 87% of men with no or mild LUTS aged 50–59, 60–69 and 70–80 years, respectively, and in 54%, 84% and 91% of men with moderate to severe LUTS
Ströberg et al. (73)	Sweden	924	Significant correlation between the IIEF-5 score and IPSS (p < 0.001) and the IIEF-5 score and LUTS-induced bother (p < 0.001)
Moreira et al. (74)	Brazil	602	LUTS were significantly (p < 0.05) associated with increased prevalence of ED
El-Sakka (75)	Egypt	476	LUTS were significantly associated with ED with a significant association between the increased severity of LUTS and the increased severity of ED
Ozayar et al. (76)	Turkey	453	ED was reported in 36% of men with moderate LUTS and in 94% with severe LUTS (p < 0.001). The odds ratio for ED was 28.7 for severe LUTS
Tsao et al. (77)	Taiwan	398	The prevalence of moderate to severe ED (IIEF-5 < 12) was significantly associated with LUTS severity (p < 0.05). A consistent inverse correlation was found between IIEF-5 and IPSS severity across the age groups, with the strongest effect observed in patients aged 60–69 years (p < 0.01)
Mehraban et al. (78)	Iran	357	Sexual dysfunction, defined as IIEF score of ≤ 20, was present in 68.2% of LUTS patients. All IIEF domain scores and the overall score were correlated with age (p < 0.001) and the IPSS (p < 0.001)
Wong et al. (79)	China	352	In multivariate analysis, moderate LUTS was independently associated with increased odds of having ED (odds ratio of 3.7, CI: 1.6–8.3)
Tsai et al. (80)	Taiwan	339	In multiple logistic regression analysis, age and IPSS (p < 0.001 and p = 0.013, respectively) were significantly associated with ED after controlling other co-morbidities
Wang et al. (81)	China	245	ED incidence significantly correlated with the severity of LUTS (p < 0.01); 71.3%, 89.6% and 95.8%, respectively, in the groups with mild, moderate and severe LUTS
Nakamura et al. (82)	Japan	220	The total IIEF-5 score significantly correlated with both the IPSS and CLSS questionnaires (p = 0.0001)
Rhoden et al. (83)	Brazil	192	Overall IPSS scores were significantly associated with ED (p = 0.002) and there was an association between the severity of ED and LUTS (p = 0.008). Logistic regression analyses showed that IPSS scores and ED remained independently associated even after controlling for confounding factors (p = 0.01)
Demir et al. (84)	Turkey	190	In the severe LUTS patient group, IIEF erectile function domain scores were significantly lower than in the moderate LUTS patient group (p < 0.05). Multiple logistic regression analysis confirmed that presence of ED was the most significant predictor of severe LUTS
Elliott et al. (85)	USA	181	A consistent negative correlation was found between obstructive IPSS and the SHIM score across age groups, with the strongest effect observed for men aged 60–70 years (p = 0.003)
Ikuerowo et al. (86)	Nigeria	132	The second question in the IIEF-5 questionnaire (How would you rate your ability to have an erection hard enough for penetration?) showed a significant correlation with total IPSS score (p = 0.022). The sum of the IPSS obstructive symptoms scores showed a significant correlation with ED scores (p < 0.001)
Glina et al. (87)	Brazil	118	Significant correlation between the IPSS and the SHIM (p < 0.001)

ED, erectile dysfunction; LUTS, lower urinary tract symptoms; BPH, benign prostatic hyperplasia; EJD, ejaculatory dysfunction; PE, painful ejaculation; CI, confidence interval; IPSS, International Prostate Symptoms Score; IIEF, International Index of Erectile Function; COPD, chronic obstructive pulmonary disease.

- They improve understanding of the aetiology of the conditions.
- They enable patients to connect conditions and risk factors.
- They can inform case finding and screening strategies.
- They can identify co-morbidities.
- They can affect the choice of appropriate treatment.

Despite the wealth of literature in support of a link between ED and LUTS, there appears to be a lack of awareness of this link in both primary and secondary care, which manifests itself in underuse of appropriate diagnostic tools (22) or prescribed drugs (23). For example, in a UK audit of 100 patients with LUTS, GPs enquired about ED in < 10% and offered no therapy to more than 80% of those with ED, yet 91% of untreated ED patients said they would like medical treatment (23). A brief survey of the services used to inform GPs in the UK shows that although the possibility of a link may be mentioned, no recommendations on co-diagnosis are given; for example, in clinical IT systems such as WebMentor, there is no mention of ED or sexual function in LUTS information for doctors and no mention of LUTS in the ED information. Similarly, in the recent NICE LUTS guidelines, although there is a mention of the association with ED, there are no constructive recommendations about how to ask about or manage any ED issues: ‘Men with lower urinary tract symptoms (LUTS) should have access to care that can help with their emotional and physical conditions and with relevant physical, psychological, sexual and social issues’ (6). In the LUTS ‘Map of Medicine’, there is a mention about enquiring about sexual function, but no mention of LUTS in the ED map (24). Several factors may reduce the likelihood of physicians asking about sexual function such as lack of time, embarrassment and insufficient knowledge on sexual health (25), but the most likely reason for failure to ask about ED in LUTS (and *vice versa*) is lack of awareness of the link between the two conditions.

Reasons why enquiries about sexual function may not be made:

- lack of time
- embarrassment
- lack of confidence
- respecting patient's privacy
- difficulty in knowing how to ask
- conservative sexual beliefs
- insufficient knowledge on sexual health
- insufficient acceptance of patient's special sexual profile

The aim of this publication was to raise awareness of the link between ED and LUTS for the purposes of possible co-diagnosis. We present a summary of the evidence for a link between ED and LUTS and highlight the need to consider both conditions together during the diagnostic process.

## Evidence in support of a link between ED and LUTS

### Epidemiological evidence

- Despite the differences in design, many large studies using well-powered multivariate analyses consistently provide overwhelming evidence of a link between ED and LUTS.

There is a wealth of published data in support of a link between ED and LUTS; Table 1 outlines the main results from larger published studies although it is not intended as a comprehensive list. Several reviews have also published tabulations of selected epidemiological studies that support the existence of a link between ED and LUTS that is independent of age or co-morbidities (26–29).

**Figure 1:**
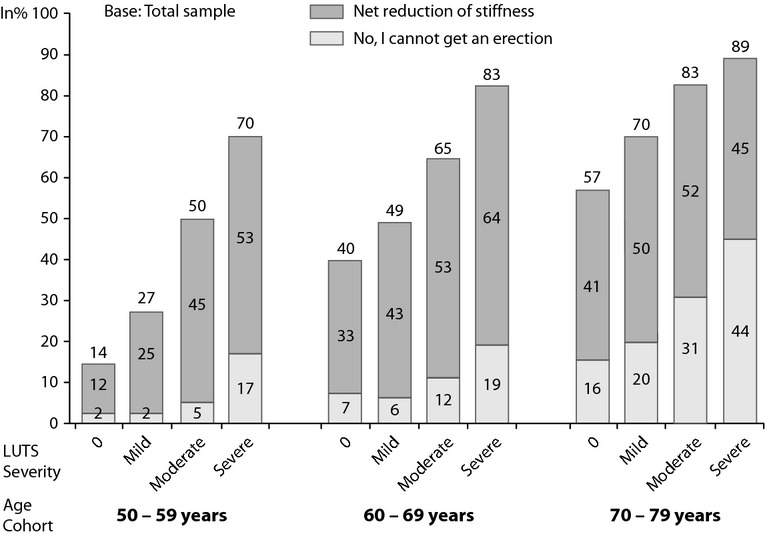
Erectile dysfunction according to age and lower urinary tract symptoms severity (30)

In some of the larger studies that have evaluated relative risk or odds ratios of the probability of a link, the results clearly highlight the association between the two conditions. For example, in the large multinational survey of the ageing male (MSAM-7) (30), multivariate analysis showed that severity of LUTS was a strong predictor of sexual dysfunction, with an odds ratio for erection problems of 8.90 (95% CI: 6.85–11.55) in those with severe LUTS. Indeed, results from this important study essentially showed that going from mild to moderate, or moderate to severe LUTS, had a greater impact on ED than ageing by 10 years (Figure 1). Similarly, in a study involving patients in UK general practices (31), odds ratios for ED in patients with both storage and voidance LUTS were calculated to be 4.0 (95% CI: 3.4–4.8). From the ED perspective, a study in Finland involving over a 1000 men (32) calculated the relative risk of LUTS in patients with severe ED as 2.3 (95% CI: 1.4–3.8). In addition to these individual studies, a recent systematic review of available epidemiological data has concluded that most men seeking treatment for either LUTS or ED have both conditions (26). Taken together, these results provide overwhelming evidence of the independent association between LUTS and ED and highlight the importance of awareness of this link for the practising physician.

### Pathogenesis (Figure 2)

- Current preclinical evidence suggests that several common pathogenetic mechanisms are involved in the development of both ED and LUTS associated with BPH.

**Figure 2:**
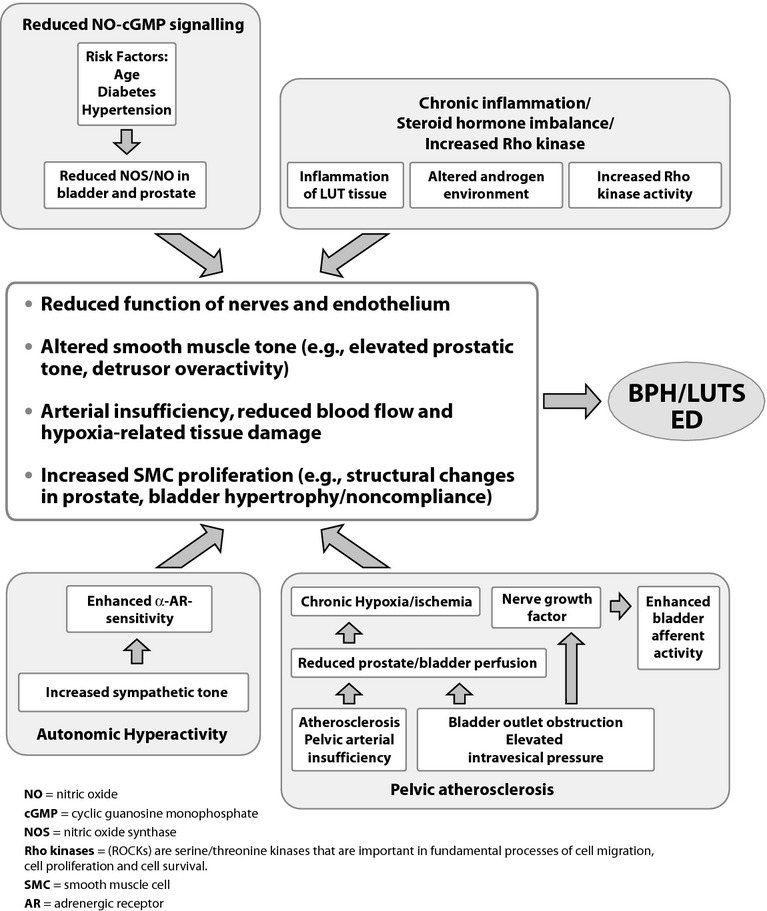
Potential pathophysiological pathways leading to lower urinary tract symptoms in men (Adapted from Refs (27,33))

The pathogenetic mechanisms underlying the relationship between LUTS and ED have been the subject of several recent reviews (27–29,33). These publications present preclinical data and well-defined theories for mechanisms currently thought to be involved, including, alteration of the nitric oxide-cyclic guanosine monophosphate pathway; enhancement of RhoA–Rho-kinase (ROCK) signalling; autonomic hyperactivity; and pelvic atherosclerosis. Additional contributing factors such as chronic inflammation (34) and sex steroid ratio imbalance may also play a role (35). Knowledge of the common pathways linking these mechanisms should allow a better understanding of the pathophysiology of both conditions (27).

## The need for co-diagnosis

**Figure 3:**
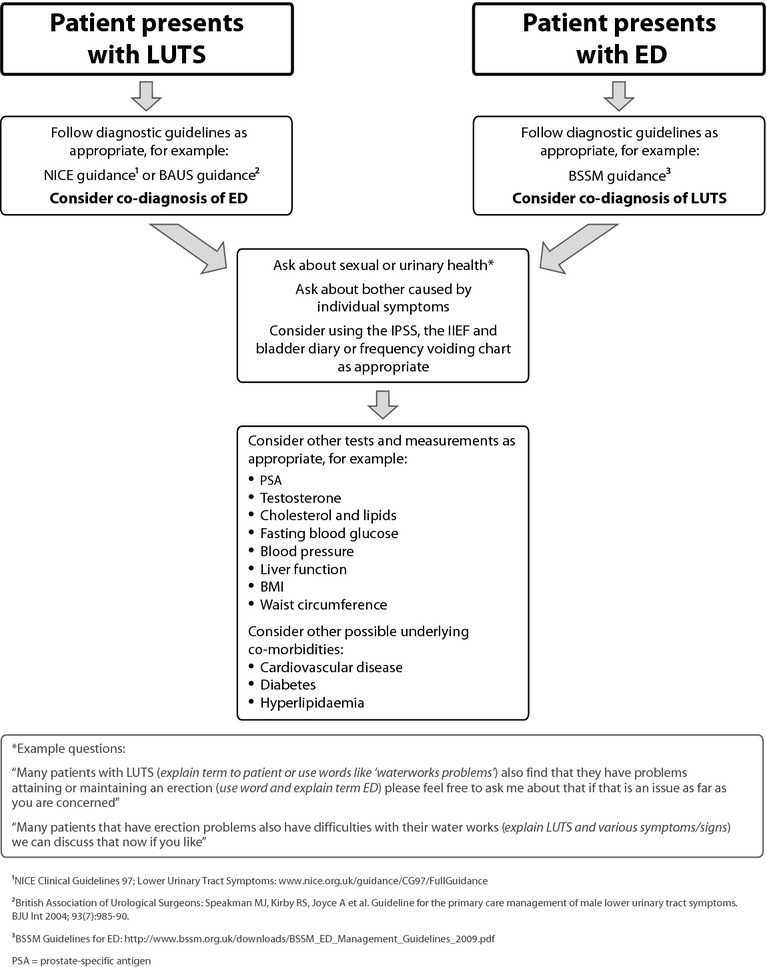
Co-diagnosis algorithm for erectile dysfunction and lower urinary tract symptoms

- Given the strong evidence from multiple epidemiological studies that ED and LUTS are correlated, independent of age or co-morbidities (Table 1), we recommend that patients seeking consultation for one condition should always be screened for complaints about the other condition.
- We propose that co-diagnosis could follow the algorithm given in Figure 3; this would then ensure that patient management accounts for all possible associated conditions.

## Key considerations

### Risk factors

- Medical, demographic and lifestyle risk factors can help to inform diagnoses and should be taken into consideration during the initial consultation.
- Awareness of risk factors may alert physicians to patients at risk of ED or LUTS and allow them to diagnose and manage both conditions appropriately.
- ED is associated with increased all-cause mortality principally because of mortality from cardiovascular disease (CVD) (36); early diagnosis of ED in patients with LUTS could help reduce the risk of subsequent CVD.
- Some LUTS in men are associated with increased all-cause mortality; in particular, older men with nocturia have an increased likelihood of CVD and mortality even after adjusting for age, body mass index and urological medications (37). Early diagnosis and management of LUTS in a patient with ED could help reduce the risk of subsequent CVD and associated early death.
- Physicians confronted with a patient with ED and/or LUTS should consider the possibility that the patient may also have type 2 diabetes mellitus (T2DM), hypertension, dyslipidaemia, other aspects of the metabolic syndrome or hypogonadism, and *vice versa*.

Risk factors are summarised in Table 2.

**Table 2:** Risk factors associated with ED and/or LUTS

Risk factor	ED	LUTS
Age	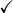 (2)	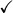 (11)
Sedentary lifestyle and lack of exercise	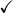 (38)	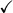 (10)*
Smoking and excessive alcohol intake	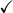 (38)	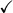 (10)*
Depression	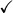 (39)	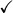 (45)
Insomnia	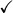 (40)	
Hypertension	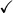 (39)	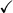 (88)
Cardiovascular disease	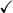 (36)	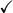 (10)*
Hyperlipidaemia	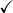 (30)	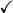 (30)
Type 2 diabetes mellitus	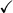 (39)	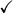 (10)*
Obesity/waist circumference	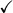 (45)	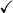 (45)
Hypogonadism	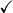 (89)	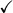 (43)
Prostate disorder	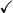 (40)	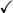 (10)*
Inflammation		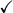 (10)*

* This is a review article that discusses the many risk factors for LUTS, citing original evidence where appropriate.

ED, erectile dysfunction; LUTS, lower urinary tract symptoms.

Several risk factors are associated with sexual dysfunction in men, including, age, individual general health status, sedentary lifestyle, depression, insomnia and other psychiatric/psychological disorders, diabetes mellitus, hypertension and CVD, hyperlipidaemia, other genitourinary disease, socio-demographic conditions and pelvic surgery (2,30,36,38,39). A recent population-based cohort study of more than 3000 older men (75–95 years, mean 82 years) from Perth, Western Australia, showed that CVD, diabetes, depression, prostate disorders and insomnia were the factors most commonly associated with sexual problems in this cohort of older men (40). Importantly, diagnosis of ED can be predictive of CAD, with a time interval for CAD risk reduction of 2–5 years (36,41). As a result, identification of ED, particularly in men < 60 years old and in those with diabetes, can be a critical first step towards cardiovascular risk detection and reduction (42).

Several risk factors are associated with LUTS in men, including, age, BPE, hypogonadism, sedentary lifestyle, depression, hypertension and CVD, hyperlipidaemia, diabetes, obesity and inflammation (10,11,30,43–45). A recent retrospective cohort study has suggested that the use of statins may delay the development of LUTS by reducing inflammation and BPE (46). The study showed that statin use was associated with a 6.5–7 year delay in the new onset of moderate to severe LUTS or BPE.

More recently, ED and LUTS have been included in the list of factors associated with metabolic syndrome (47); specifically, waist circumference has been significantly and positively associated with prostate volume, serum prostate-specific antigen and international prostate symptoms score (45). Higher waist circumferences have also been significantly associated with a greater prevalence of hypertension, CAD, T2DM and obesity, as well as the presence of ED and ejaculatory dysfunction (45). Metabolic syndrome is defined by a cluster of findings that directly increase the risk of CVD and T2DM. The main components are dyslipidaemia, hypertension and dysregulated glucose homeostasis, but central obesity (waist circumference) and/or insulin resistance are receiving increasing attention as the core manifestations of the syndrome (48). Other abnormalities such as chronic pro-inflammatory and pro-thrombotic states, non-alcoholic fatty liver disease and sleep apnoea have also recently been added to the spectrum, making the definition of metabolic syndrome even more complex (48). The most relevant current definition incorporates the International Diabetes Federation (IDF) and American Heart Association/National Heart, Lung and Blood Institute (AHA/NHLBI) definitions and requires a patient to have any three of the following conditions (49).^2^

- Elevated waist circumference (ethnicity specific values, e.g. for European males > 94 cm and females > 80 cm).
- Triglycerides 1.7 mmol/l or greater (> 50 mg/dl).
- HDL-cholesterol below 1.03 mmol/l (< 40 mg/dl) in males and below 1.29 mmol/l (< 50 mg/dl) in females.
- BP 130/85 mmHg or greater.
- Fasting glucose 5.6 mmol/l or greater (> 100 mg/dl).

### Management issues

- Prescribing physicians should be aware of the sexual adverse effects of treatments for LUTS; by thinking about co-diagnosis of ED, sexual function will be fully evaluated prior to commencement of treatment and monitored throughout to ensure that the choice of drug treatment is appropriate.
- Physicians should make patients aware of the potential sexual adverse effects of some treatments prior to prescribing; this may be of particular relevance in younger patients.
- Physicians should also assess co-morbidities, concomitant medications and lifestyle to consider all associated risk factors and manage accordingly.

Many therapies recommended for the treatment of LUTS can affect sexual function and there are known cases of effects such as loss of libido, ED and ejaculatory disorders (Table 3) (27,50). The alpha(1)-blockers alfuzosin, doxazosin and terazosin appear to be associated with fewer sexual adverse effects than the alpha(1)-blocker tamsulosin or 5-alpha-reductase inhibitors, and alpha(1)-blocker plus 5-alpha-reductase inhibitor combination therapy (51). The newer alpha-blocker, silodosin may be associated with a higher incidence of sexual side effects than other drugs in the same class; a recent clinical trial investigating the effects of silodosin therapy for LUTS in men with suspected BPH–LUTS reported the percentage of subjects reporting ‘retrograde ejaculation’ was 14.2% in the silodosin group, which was significantly higher than the 2.1% and 1.1% in the tamsulosin and placebo treatment groups, respectively (52). Combination therapy is likely to increase the risk of sexual adverse effects (50), for example, in the MTOPS study (Medical Therapy of Prostatic Symptoms) (53), ED was volunteered as a side effect in 5.8% of patients treated with the combination of finasteride and doxazosin, a higher proportion than seen with either agent alone. However, it is worth noting that there was no baseline assessment of sexual function in this study and as a result, these data could be misleading.

**Table 3:** Sexual adverse events associated with drugs used in the treatment for BPH–LUTS reported from clinical trials (Adapted from Refs (50,52))

	ED (%)	EJD (%)	Retrograde ejaculation*(%)	Decreased libido (%)
**Alpha-blockers**				
Alfuzosin	3	–		1
Doxazosin	4	0		3
Tamsulosin	4	10	2.1*	–
Terazosin	5	1		3
Placebo	4	1	1.1*	3
Silodosin	–	28†	14.2*	–
**Dutasteride**‡				
Dutasteride	7.3	2.2		4.2
Placebo	4.0	0.8		2.1
**Finasteride**‡				
Finasteride§	8.1	4.5		6.4
Placebo§	3.7	0.9		3.4

* Taken from Chapple (52).

† Treatment-emergent adverse events. Data from pooled results of two phase III, 12-week studies.

‡ Drug-related adverse events reported in year 1 of study.

§ EJD includes decreased ejaculate volume and ejaculation disorder; ED reported as ‘impotence’. ED, erectile dysfunction; EJD, ejaculatory dysfunction; retrograde ejaculation, orgasm with no semen, orgasm semen quantity reduced, and retrograde ejaculation.

Although adverse events data from clinical trials can give an estimation of the risk of these events, it is important to tailor treatment and management individually; the incidence of adverse effects reported in clinical trials does not necessarily represent real-life experience and assessed adverse events are not necessarily representative of patient-volunteered side effects.

Many men with LUTS and/or ED will also have other health problems and may be receiving treatment with drugs that have associated sexual adverse events; for example, many antihypertensive drugs such as the diuretics can cause ED (54). Other classes of drugs that can cause sexual adverse effects include antipsychotic agents (55), antidepressants (55), beta-blockers (54), antihistamines, Parkinson's disease medications and muscle relaxants (50,56).

Attention to lifestyle modifications such as weight loss, increased physical activity and decreased use of recreational drugs including alcohol is also important, because recent research has suggested that lifestyle and metabolic factors are significantly associated with increased risks of LUTS (57). Factors associated with decreased risks include increased physical activity, moderate alcohol intake and increased vegetable consumption (58).

### Potential situations in which to refer to secondary care

- Most patients with LUTS and ED can be managed by the primary care physician. Situations in which specialist assessment may be required are outlined in current guidelines (6,59). Generally, referral will only be necessary for patients who fail to respond to initial treatment or where there are complications.

Most patients with LUTS, ED or both conditions are suitable for initial management in primary care; because these patients often have co-morbid conditions such as CVD or diabetes; the primary care physician is perfectly placed to initiate holistic assessment and management. Possible exceptions include those in whom a phosphodiesterase-5 (PDE-5) inhibitor is contraindicated, for example, when a patient has complex cardiovascular co-morbidity. Otherwise, referral need only take place for those patients who fail to respond to initial medical management in primary care or who have complications as outlined in recent guideline statements (6), or when the physician is concerned and needs further support.

Specialist assessment can be undertaken by physicians working in secondary care, or those in primary care with specialist expertise and ability for this kind of evaluation (6). NICE guidelines for LUTS recommend specialist referral when patients have bothersome LUTS that have not responded to conservative management or drug treatment, or if they have LUTS complicated by recurrent or persistent urinary tract infection, retention, renal impairment that is suspected to be caused by lower urinary tract dysfunction, or suspected urological cancer (6). Additional trigger points that may suggest a need for review or specialist referral include macroscopic haematuria, deterioration in kidney function or an unexplained, clinically significant increase in postvoid residual urine volume (60).

Current guidelines for ED in older men suggest referral be considered for men with a history of trauma (e.g. to the genital area, pelvis or spine), for men who do not respond to at least two different PDE-5 inhibitors at the maximum tolerated dose, or who have hypogonadism (59). All men with ED should have their cardiovascular risk assessed in primary care and addressed appropriately. Men with symptoms of CVD that restrict their ability to exercise need further evaluation to establish the safety of sexual activity and ED treatment. Men in their 40s and 50s who are at increased risk of cardiac events, whether cardiac symptoms are present or not, should be considered for more detailed cardiac evaluation (36,41).

Referral to secondary care should be considered when:

There is an indication for surgery

There is a complex cardiovascular co-morbidity

There is no response to conservative management or drug treatment

There is evidence of worsening on drug treatment

There is a history of trauma or abnormalities of the penis or testes

The diagnosis is complicated by symptoms that are suggestive of:

Recurrent or persistent urinary tract infection
Chronic urinary retention*
Renal impairment
Lower urinary tract disease, bladder or prostate** *cancer*

*Postvoid residual volume should be quantified when incomplete bladder emptying is suspected; although there is no consensus and considerable controversy surrounding what constitutes a diagnosis of retention, a postvoid residual volume of > 300 ml is generally agreed to suggest retention (61)

**PSA testing can be offered at the physician's discretion if symptoms are suggestive of benign prostate enlargement, the prostate feels abnormal, or the patient or physician is concerned about prostate cancer

## Conclusion

Erectile dysfunction and LUTS are conditions with both high prevalence and significant negative impact on patients' quality of life. There is a strong epidemiological evidence for a link between ED and LUTS supported by theories for their shared pathogenesis.

Although physicians are aware of LUTS and ED, knowledge that there are links between the two is often lacking. This leads to missed opportunities to diagnose and treat these conditions and to consider potential serious co-morbidities. As LUTS and ED have complex multifactorial aetiologies and multiple associations, including, diabetes, lipid disorders, metabolic syndrome and major cardiac diseases, they provide an important opportunity for interdisciplinary care and for primary care physicians to work closely with urologists, cardiologists, endocrinologists and physicians caring for elderly people.

When seeing patients with either LUTS or ED, the routine approach should be to ask about symptoms of both conditions at first contact. The management of LUTS and ED is as much about managing their associations as the prescribing of effective medication; many risk factors are shared and these need to be assessed and addressed to treat the patient holistically. For example, early diagnosis of ED in a patient presenting with LUTS and modification of any risk factors wherever possible could reduce the likelihood or delay the onset of subsequent CVD. There is clear evidence that effective treatment for one condition can improve the other, but only by assessing the co-morbidities for both conditions can therapy be tailored to achieve the desired outcome for the patient and his partner. The ability to treat both LUTS and ED together with one medication and lifestyle advice is worthy of consideration.

## Footnotes

1 ‘Benign prostatic hyperplasia’ should be reserved for histopathologically confirmed hyperplastic changes (i.e. abnormality/changes at the cell level) in the prostate (NICE 2010).

2 The IDF/AHA/NHLBI definition is given in US units, these have been converted to UK units for the purposes of this publication with the US units in brackets. In some cases, the individual values that are part of this overall definition of metabolic syndrome are a little lower than those accepted in other individual diagnoses.

## Supporting Information

**Data S1.** Online extra slideshow.

## References

[1] Hatzimouratidis K, Amar E, Eardley I (2010). Guidelines on male sexual dysfunction: erectile dysfunction and premature ejaculation. Eur Urol.

[2] Prins J, Blanker MH, Bohnen AM (2002). Prevalence of erectile dysfunction: a systematic review of population-based studies. Int J Impot Res.

[3] Braun M, Wassmer G, Klotz T (2000). Epidemiology of erectile dysfunction: results of the ‘Cologne Male Survey’. Int J Impot Res.

[4] Boyle P, Robertson C, Mazzetta C (2003). The association between lower urinary tract symptoms and erectile dysfunction in four centres: the UrEpik study. BJU Int.

[5] Inman BA, St Sauver JL, Jacobson DJ (2009). A population-based, longitudinal study of erectile dysfunction and future coronary artery disease. Mayo Clin Proc.

[6] NICE clinical guideline 97 http://www.nice.org.uk/guidance/CG97/FullGuidance.

[7] Glasser DB, Carson C, Kang JH, Laumann EO (2007). Prevalence of storage and voiding symptoms among men aged 40 years and older in a US population-based study: results from the Male Attitudes Regarding Sexual Health study. Int J Clin Pract.

[8] Sexton CC, Coyne KS, Kopp ZS (2009). The overlap of storage, voiding and postmicturition symptoms and implications for treatment seeking in the USA, UK and Sweden. EpiLUTS BJU Int.

[9] Roosen A, Chapple CR, Dmochowski RR (2009). A refocus on the bladder as the originator of storage lower urinary tract symptoms: a systematic review of the latest literature. Eur Urol.

[10] Parsons JK (2010). Benign prostatic hyperplasia and male lower urinary tract symptoms: epidemiology and risk factors. Curr Bladder Dysfunct Rep.

[11] Verhamme KM, Dieleman JP, Bleumink GS (2002). Pan European Expert Panel. Incidence and prevalence of lower urinary tract symptoms suggestive of benign prostatic hyperplasia in primary care – the triumph project. Eur Urol.

[12] Wei JT, Calhoun E, Jacobsen SJ (2005). Urologic diseases in America project: benign prostatic hyperplasia. J Urol.

[13] Kupelian V, Wei JT, O'Leary MP (2006). Prevalence of lower urinary tract symptoms and effect on quality of life in a racially and ethnically diverse random sample: the Boston Area Community Health (BACH) survey. Arch Intern Med.

[14] Taylor BC, Wilt TJ, Fink HA (2006). Prevalence, severity and health correlates of lower urinary tract symptoms among older men: the MrOS study. Urology.

[15] Parsons JK, Bergstrom J, Silberstein J, Barrett-Connor E (2008). Prevalence and characteristics of lower urinary tract symptoms in men aged > or = 80 years. Urology.

[16] van Moorselaar J (2003). LUTS and sexual dysfunction: implications for management of BPH. Eur Urol Suppl.

[17] Sand MS, Fisher W, Rosen R (2008). Erectile dysfunction and constructs of masculinity and quality of life in the multinational men's attitudes to life events and sexuality (MALES) study. J Sex Med.

[18] Fisher WA, Rosen RC, Eardley I (2005). Sexual experience of female partners of men with erectile dysfunction: the female experience of men's attitudes to life events and sexuality (FEMALES) study. J Sex Med.

[19] Wein AJ, Coyne KS, Tubaro A (2009). The impact of lower urinary tract symptoms on male sexual health: EpiLUTS. BJU Int.

[20] Mitropoulus D, Anastasiou I, Giannopouluo C (2002). Symptomatic benign prostate hyperplasia: impact on partners' quality of life. Eur Urol.

[21] Sells H, Donovan J, Ewings P, MacDonagh RP (2000). The development and validation of a quality-of-life measure to assess partner morbidity in benign prostatic hyperplasia. BJU Int.

[22] Seftel AD, Rosen RC, Rosenberg MT, Sadovsky R (2008). Benign prostatic hyperplasia evaluation, treatment and association with sexual dysfunction: practice patterns according to physician specialty. Int J Clin Pract.

[23] Chitale S, Collins R, Hull S, Smith E, Irving S (2007). Is the current practice providing an integrated approach to the management of LUTS and ED in primary care? An audit and literature review. J Sex Med.

[24] Map of Medicine (2012). http://www.mapofmedicine.com.

[25] Merrill JM, Laux LF, Thornby JI (1990). Why doctors have difficulty with sex histories. South Med J.

[26] Seftel AD, de la Rosette J, Birt J (2013). Coexisting lower urinary tract symptoms and erectile dysfunction: a systematic review of epidemiological data. Int J Clin Pract.

[27] Gacci M, Eardley I, Giuliano F (2011). Critical analysis of the relationship between sexual dysfunctions and lower urinary tract symptoms due to benign prostatic hyperplasia. Eur Urol.

[28] McVary KT (2005). Erectile dysfunction and lower urinary tract symptoms secondary to BPH. Eur Urol.

[29] Kohler TS, McVary KT (2009). The relationship between erectile dysfunction and lower urinary tract symptoms and the role of phosphodiesterase type 5 inhibitors. Eur Urol.

[30] Rosen R, Altwein J, Boyle P (2003). Lower urinary tract symptoms and male sexual dysfunction: the multinational survey of the aging male (MSAM-7). Eur Urol.

[31] Morant S, Bloomfield G, Vats V, Chapple C (2009). Increased sexual dysfunction in men with storage and voiding lower urinary tract symptoms. J Sex Med.

[32] Shiri R, Häkkinen J, Koskimäki J (2007). Erectile dysfunction influences the subsequent incidence of lower urinary tract symptoms and bother. Int J Impot Res.

[33] Andersson KE, de Groat WC, McVary KT (2011). Tadalafil for the treatment of lower urinary tract symptoms secondary to benign prostatic hyperplasia: pathophysiology and mechanism(s) of action. Neurourol Urodyn.

[34] Penna G, Fibbi B, Amuchastegui S (2009). Human benign prostatic hyperplasia stromal cells as inducers and targets of chronic immuno-mediated inflammation. J Immunol.

[35] Corona G, Maggi M (2010). The role of testosterone in erectile dysfunction. Nat Rev Urol.

[36] Jackson G, Boon N, Eardley I (2010). Erectile dysfunction and coronary artery disease prediction: evidence-based guidance and consensus. Int J Clin Pract.

[37] Lightner DJ, Krambeck AE, Jacobson DJ (2012). Nocturia is associated with an increased risk of coronary heart disease and death. BJU Int.

[38] Kupelian V, Araujo AB, Gretchen R (2010). Relative contributions of modifiable risk factors to erectile dysfunction. Results from the Boston Area Community Health (BACH) Survey. Prev Med.

[39] Feldman HA, I Goldstein, DG Hatzichristou (1994). Impotence and its medical and psychosocial correlates: results of the Massachusetts Male Aging Study. J Urol.

[40] Hyde Z, Flicker L, Hankey GJ (2012). Prevalence and predictors of sexual problems in men aged 75–95 years: a population-based study. J Sex Med.

[41] Nehra A, Jackson G, Miner M (2012). The Princeton III consensus recommendations for the management of erectile dysfunction and cardiovascular disease. Mayo Clin Proc.

[42] Miner M, Seftel AD, Nehra A (2012). Prognostic utility of erectile dysfunction for cardiovascular disease in younger men and those with diabetes. Am Heart J.

[43] Favilla V, Cimino S, Castelli T (2010). Relationship between lower urinary tract symptoms and serum levels of sex hormones in men with symptomatic benign prostatic hyperplasia. BJU Int.

[44] Lee SH, Lee WK, Lee SK (2011). The association between lower urinary tract symptoms and depression in aging men: Hallym Aging Study. Eur Urol Suppl.

[45] Lee RK, Chung D, Chughtai B (2012). Central obesity as measured by waist circumference is predictive of severity of lower urinary tract symptoms. BJU Int.

[46] St Sauver JL, Jacobsen SJ, Jacobson DJ (2011). Statin use and decreased risk of benign prostatic enlargement and lower urinary tract symptoms. BJU Int.

[47] Hammarsten J, Peeker R (2011). Urological aspects of the metabolic syndrome. Nat Rev Urol.

[48] Kassi E, Pervanidou P, Kaltsas G, Chrousos G (2011). Metabolic syndrome: definitions and controversies. BMC Med.

[49] Alberti KG, Eckel RH, Grundy SM (2009). International Diabetes Federation Task Force on Epidemiology and Prevention, National Heart, Lung and Blood Institute, American Heart Association, World Heart Federation, International Atherosclerosis Society, International Association for the Study of Obesity: harmonizing the metabolic syndrome: a joint interim statement of the International Diabetes Federation Task Force on Epidemiology and Prevention; National Heart, Lung, and Blood Institute; American Heart Association; World Heart Federation; International Atherosclerosis Society; and International Association for the Study of Obesity. Circulation.

[50] Mirone M, Sessa A, Giuliano F (2011). Current benign prostatic hyperplasia treatment: impact on sexual function and management of related sexual adverse events. Int J Clin Pract.

[51] Rosen RC, Link CL, O'Leary MP (2009). Lower urinary tract symptoms and sexual health: the role of gender, lifestyle and medical co-morbidities. BJU Int.

[52] Chapple CR, Montorsi F, Tammela TLJ (2011). Silodosin therapy for lower urinary tract symptoms in men with suspected benign prostatic hyperplasia: results of an international, randomized, double-blind, placebo- and active-controlled clinical trial performed in Europe. Eur Urol.

[53] McConnell JD, Roehrborn CG, Bautista OM (2003). The long-term effect of doxazosin, finasteride and combination therapy on the clinical progression of benign prostatic hyperplasia. N Engl J Med.

[54] Ferrario CM, Levy P (2002). Sexual dysfunction in patients with hypertension: implications for therapy. J Clin Hypertens.

[55] Baldwin D, Mayers A (2003). Antipsychotics and antidepressants sexual side-effects of antidepressant and antipsychotic drugs. Adv Psychiatr Treat.

[56] WebMD Erectile Dysfunction Health Centre (2012). http://www.webmd.com/erectile-dysfunction/guide/drugs-linked-erectile-dysfunction.

[57] Parsons JK, Messer K, White M (2011). Obesity increases and physical activity decreases lower urinary tract symptom risk in older men: the Osteoporotic Fractures in Men Study. Eur Urol.

[58] Parsons JK (2011). Lifestyle factors, benign prostatic hyperplasia and lower urinary tract symptoms. Curr Opin Urol.

[59] Hackett G, Kell P, Ralph D (2008). British Society for Sexual Medicine guidelines on the management of erectile dysfunction. J Sex Med.

[60] Oelke M, Burger M, Castro-Diaz D (2011). Diagnosis and medical treatment of lower urinary tract symptoms in adult men: applying specialist guidelines in clinical practice. BJU Int.

[61] Gujral S, Abrams P, Donovan JL (2000). A prospective randomized trial comparing transurethral resection of the prostate and laser therapy in men with chronic urinary retention: the CLasP study. J Urol.

[62] McVary K, Foley KA, Long SR (2008). Identifying patients with benign prostatic hyperplasia through a diagnosis of, or treatment for, erectile dysfunction. Curr Med Res Opin.

[63] Rosen RC, Wei JT, Althof SE (2009). BPH Registry and Patient Survey Steering Committee. Association of sexual dysfunction with lower urinary tract symptoms of BPH and BPH medical therapies: results from the BPH Registry. Urology.

[64] Blanker MH, Bohnen AM, Groeneveld FPMJ (2001). Correlates for erectile and ejaculatory dysfunction in older Dutch men: a community-based study. J Am Geriatr Soc.

[65] Hansen BL (2004). Lower urinary tract symptoms (LUTS) and sexual function in both sexes. Eur Urol.

[66] Ponholzer A, Temml C, Obermayr R, Madersbacher S (2004). Association between lower urinary tract symptoms and erectile dysfunction. Urology.

[67] Chung WS, Nehra A, Jacobson DJ (2004). Lower urinary tract symptoms and sexual dysfunction in community-dwelling men. Mayo Clin Proc.

[68] Macfarlane GJ, Botto H, Sagnier PP (1996). The relationship between sexual life and urinary condition in the French community. J Clin Epidemiol.

[69] Frankel SJ, Donovan JL, Peters TI (1998). Sexual function in men with lower urinary tract symptoms. J Clin Epidemiol.

[70] Song J, Shao Q, Tian Y (2011). Association between lower urinary tract symptoms and erectile dysfunction in males aged 50 years and above: results from a multicenter community-based cross-sectional survey (BPC-BPH). Zhonghua Yi Xue Za Zhi.

[71] Vallancien G, Emberton M, Harving N, van Moorselaar J, for the ALFONE Study Group (2003). Sexual dysfunction in 1,274 European men suffering from lower urinary tract symptoms. J Urol.

[72] Li MK, Garcia LA, Rosen R (2005). Lower urinary tract symptoms and male sexual dysfunction in Asia: a survey of aging men from five Asian countries. BJU Int.

[73] Ströberg P, Boman H, Gellerstedt M, Hedelin H (2006). Relationships between lower urinary tract symptoms, the bother they induce and erectile dysfunction. Scand J Urol Nephrol.

[74] Moreira ED, Lisboa Lobo CF, Villa M, Nicolosi A, Glasser DB (2002). Prevalence and correlates of erectile dysfunction in Salvador, northeastern Brazil: a population-based study. Int J Impot Res.

[75] El-Sakka AI (2006). Lower urinary tract symptoms in patients with erectile dysfunction: analysis of risk factors. J Sex Med.

[76] Ozayar A, Zumrutbas AE, Yaman O (2008). The relationship between lower urinary tract symptoms (LUTS), diagnostic indicators of benign prostatic hyperplasia (BPH), and erectile dysfunction in patients with moderate to severely symptomatic BPH. Int Urol Nephrol.

[77] Tsao CW, Cha TL, Lee SS (2008). Association between lower urinary tract symptoms and sexual dysfunction in Taiwanese men. Andrologia.

[78] Mehraban D, Naderi GH, Yahyazadeh SR, Amirchaghmaghi M (2008). Sexual dysfunction in aging men with lower urinary tract symptoms. Urol J.

[79] Wong SY, Chan D, Hong A (2006). Depression and lower urinary tract symptoms: two important correlates of erectile dysfunction in middle-aged men in Hong Kong. China Int J Urol.

[80] Tsai CC, Liu CC, Huang SP (2010). The impact of irritative lower urinary tract symptoms on erectile dysfunction in aging Taiwanese males. Aging Male.

[81] Wang Y, Sun GF, He LJ (2008). Correlation of lower urinary tract symptoms with erectile dysfunction in men aged 50 years and above. Zhonghua Nan Ke Xue.

[82] Nakamura M, Fujimura T, Nagata M (2012). Association between lower urinary tract symptoms and sexual dysfunction assessed using the core lower urinary tract symptom score and International index of erectile function-5 questionnaires. Aging Male.

[83] Rhoden EL, Riedner CE, Fornari A (2008). Evaluation of the association between lower urinary tract symptoms and erectile dysfunction, considering its multiple risk factors. J Sex Med.

[84] Demir O, Akgul K, Akar Z (2009). Association between severity of lower urinary tract symptoms, erectile dysfunction and metabolic syndrome. Aging Male.

[85] Elliott SP, Gulati M, Pasta DJ (2004). Obstructive lower urinary tract symptoms correlate with erectile dysfunction. Urology.

[86] Ikuerowo SO, Akindiji YO, Akinoso OA (2008). Association between erectile dysfunction and lower urinary tract symptoms due to benign prostatic hyperplasia in Nigerian men. Urol Int.

[87] Glina S, Santana AW, Azank F (2006). Lower urinary tract symptoms and erectile dysfunction are highly prevalent in ageing men. BJU Int.

[88] Sugaya K, Kadekawa K, Ikehara A (2003). Influence of hypertension on lower urinary tract symptoms in benign prostatic hyperplasia. Int J Urol.

[89] Hwang TI, Lin YC (2008). The relationship between hypogonadism and erectile dysfunction. Int J Impot Res.

