# Supplementary material for: Erectile dysfunction and lower urinary tract symptoms: a consensus on the importance of co-diagnosis
Source: Int J Clin Pract. 2013 Apr 25;67(7):606–18. doi: 10.1111/ijcp.12176 (PMC3748789; doi:10.1111/ijcp.12176)
Supplement: Supplementary file 1 [file ijcp0067-0606-SD1.ppt]

## Slide 1
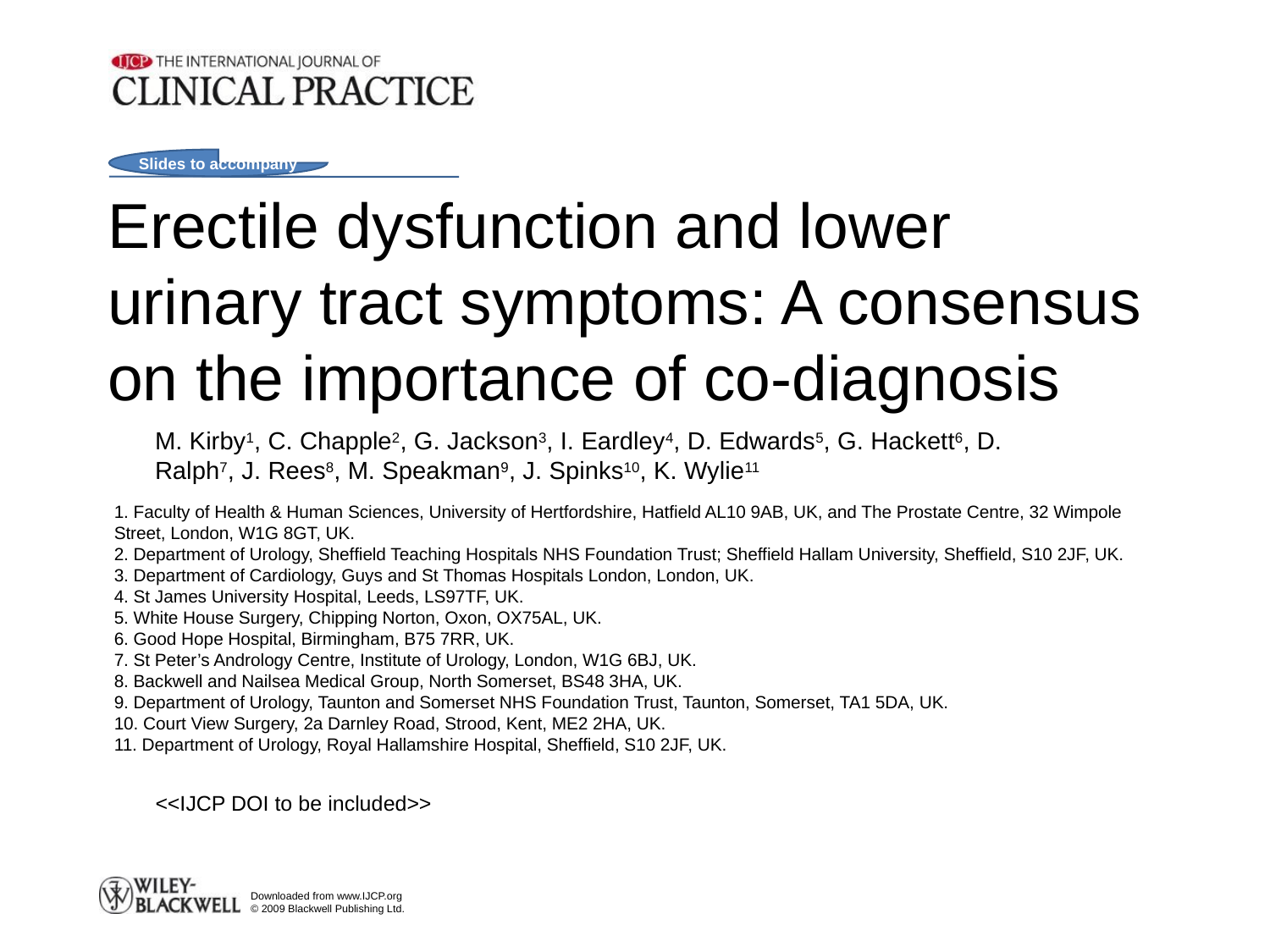

# Erectile dysfunction and lower urinary tract symptoms: A consensus on the importance of co-diagnosis
M. Kirby1, C. Chapple2, G. Jackson3, I. Eardley4, D. Edwards5, G. Hackett6, D. Ralph7, J. Rees8, M. Speakman9, J. Spinks10, K. Wylie11
1. Faculty of Health & Human Sciences, University of Hertfordshire, Hatfield AL10 9AB, UK, and The Prostate Centre, 32 Wimpole Street, London, W1G 8GT, UK.
2. Department of Urology, Sheffield Teaching Hospitals NHS Foundation Trust; Sheffield Hallam University, Sheffield, S10 2JF, UK.
3. Department of Cardiology, Guys and St Thomas Hospitals London, London, UK.
4. St James University Hospital, Leeds, LS97TF, UK.
5. White House Surgery, Chipping Norton, Oxon, OX75AL, UK.
6. Good Hope Hospital, Birmingham, B75 7RR, UK.
7. St Peter’s Andrology Centre, Institute of Urology, London, W1G 6BJ, UK.
8. Backwell and Nailsea Medical Group, North Somerset, BS48 3HA, UK.
9. Department of Urology, Taunton and Somerset NHS Foundation Trust, Taunton, Somerset, TA1 5DA, UK.
10. Court View Surgery, 2a Darnley Road, Strood, Kent, ME2 2HA, UK.
11. Department of Urology, Royal Hallamshire Hospital, Sheffield, S10 2JF, UK.
<<IJCP DOI to be included>>

## Slide 2
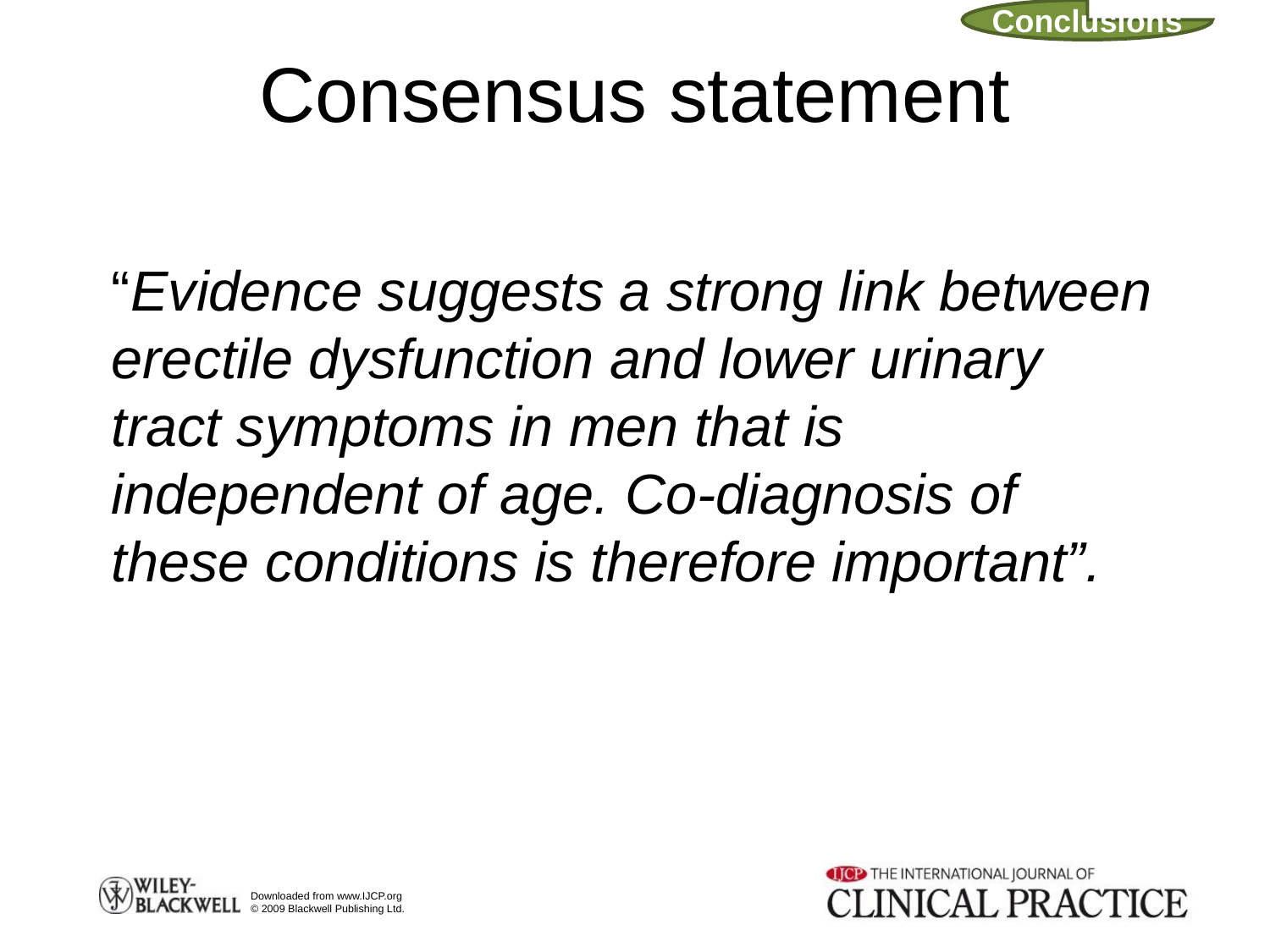

# Consensus statement
“Evidence suggests a strong link between erectile dysfunction and lower urinary tract symptoms in men that is independent of age. Co-diagnosis of these conditions is therefore important”.

## Slide 3
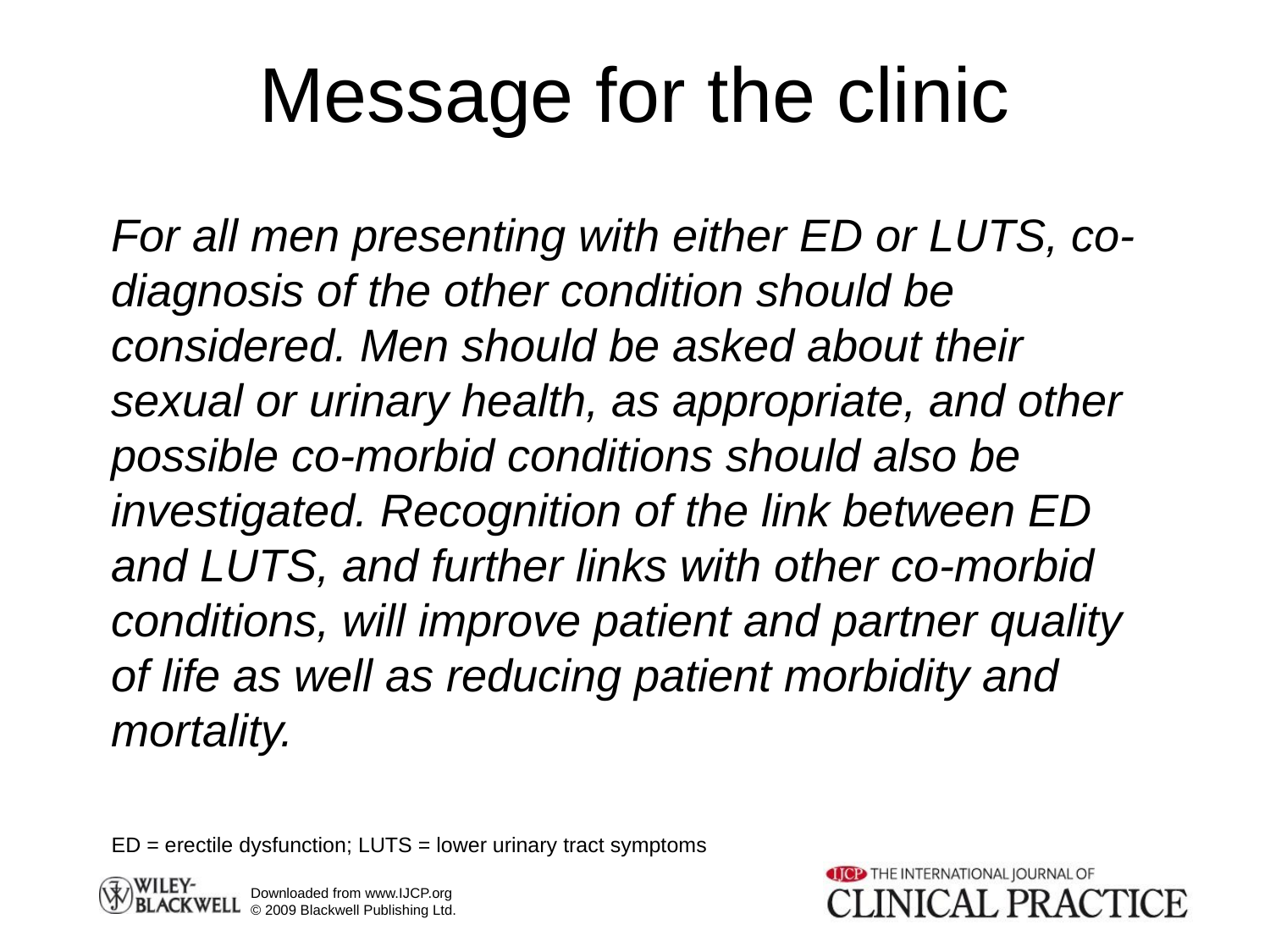

# Message for the clinic
For all men presenting with either ED or LUTS, co-diagnosis of the other condition should be considered. Men should be asked about their sexual or urinary health, as appropriate, and other possible co-morbid conditions should also be investigated. Recognition of the link between ED and LUTS, and further links with other co-morbid conditions, will improve patient and partner quality of life as well as reducing patient morbidity and mortality.
ED = erectile dysfunction; LUTS = lower urinary tract symptoms

## Slide 4
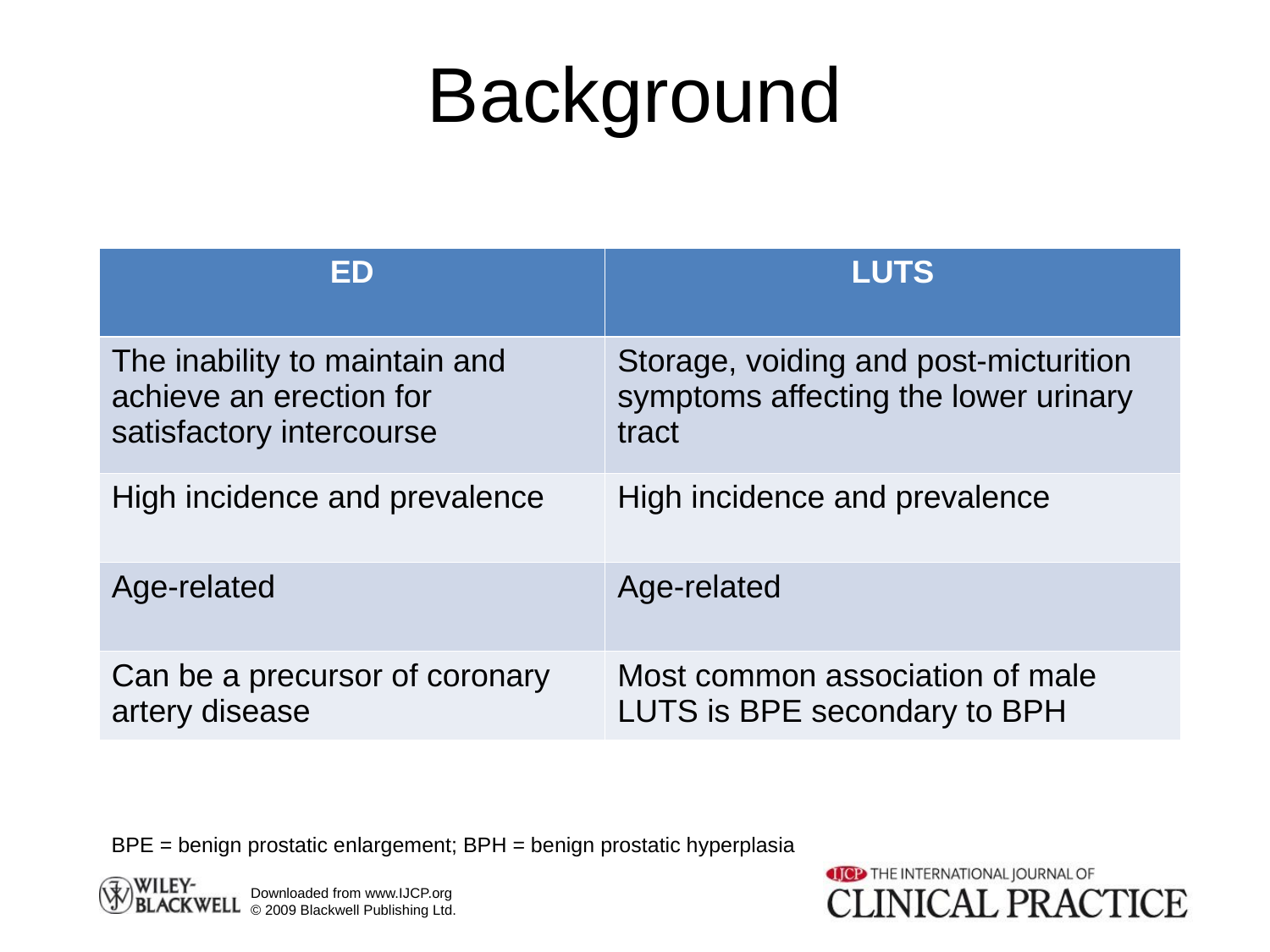

# Background
| ED | LUTS |
| --- | --- |
| The inability to maintain and achieve an erection for satisfactory intercourse | Storage, voiding and post-micturition symptoms affecting the lower urinary tract |
| High incidence and prevalence | High incidence and prevalence |
| Age-related | Age-related |
| Can be a precursor of coronary artery disease | Most common association of male LUTS is BPE secondary to BPH |
BPE = benign prostatic enlargement; BPH = benign prostatic hyperplasia

## Slide 5
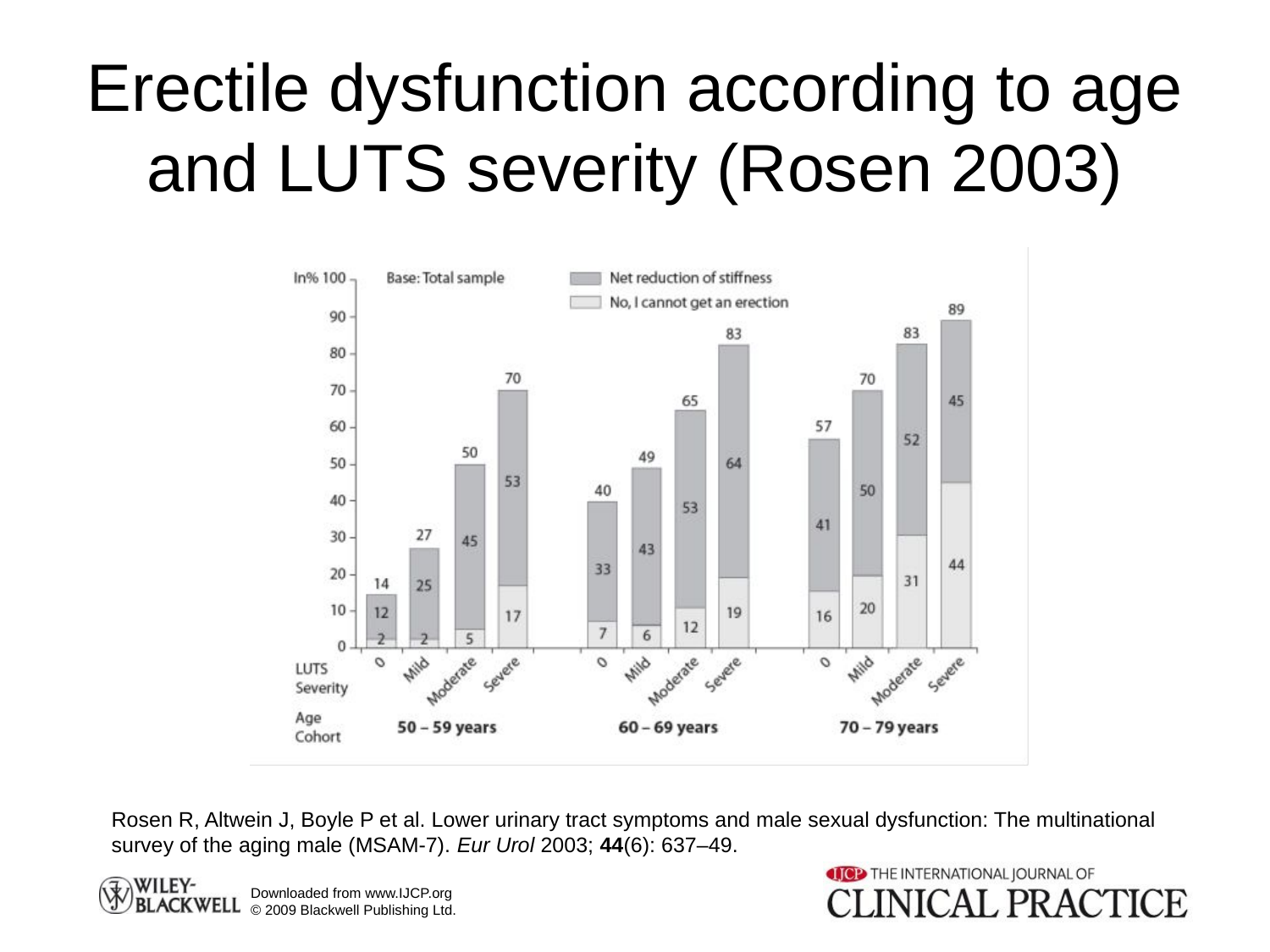

# Erectile dysfunction according to age and LUTS severity (Rosen 2003)
Rosen R, Altwein J, Boyle P et al. Lower urinary tract symptoms and male sexual dysfunction: The multinational survey of the aging male (MSAM-7). Eur Urol 2003; 44(6): 637–49.

## Slide 6
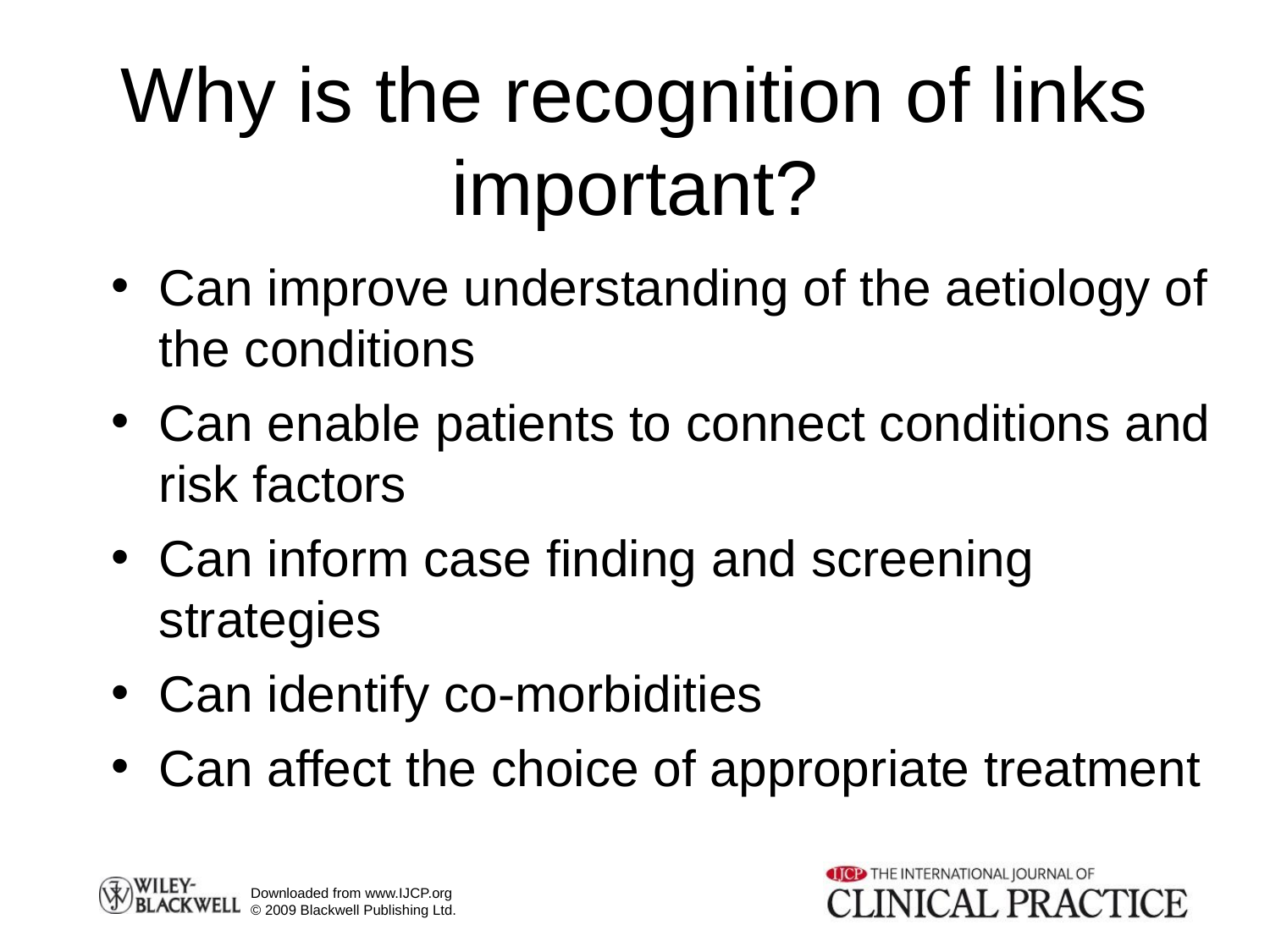

# Why is the recognition of links important?
Can improve understanding of the aetiology of the conditions
Can enable patients to connect conditions and risk factors
Can inform case finding and screening strategies
Can identify co-morbidities
Can affect the choice of appropriate treatment

## Slide 7
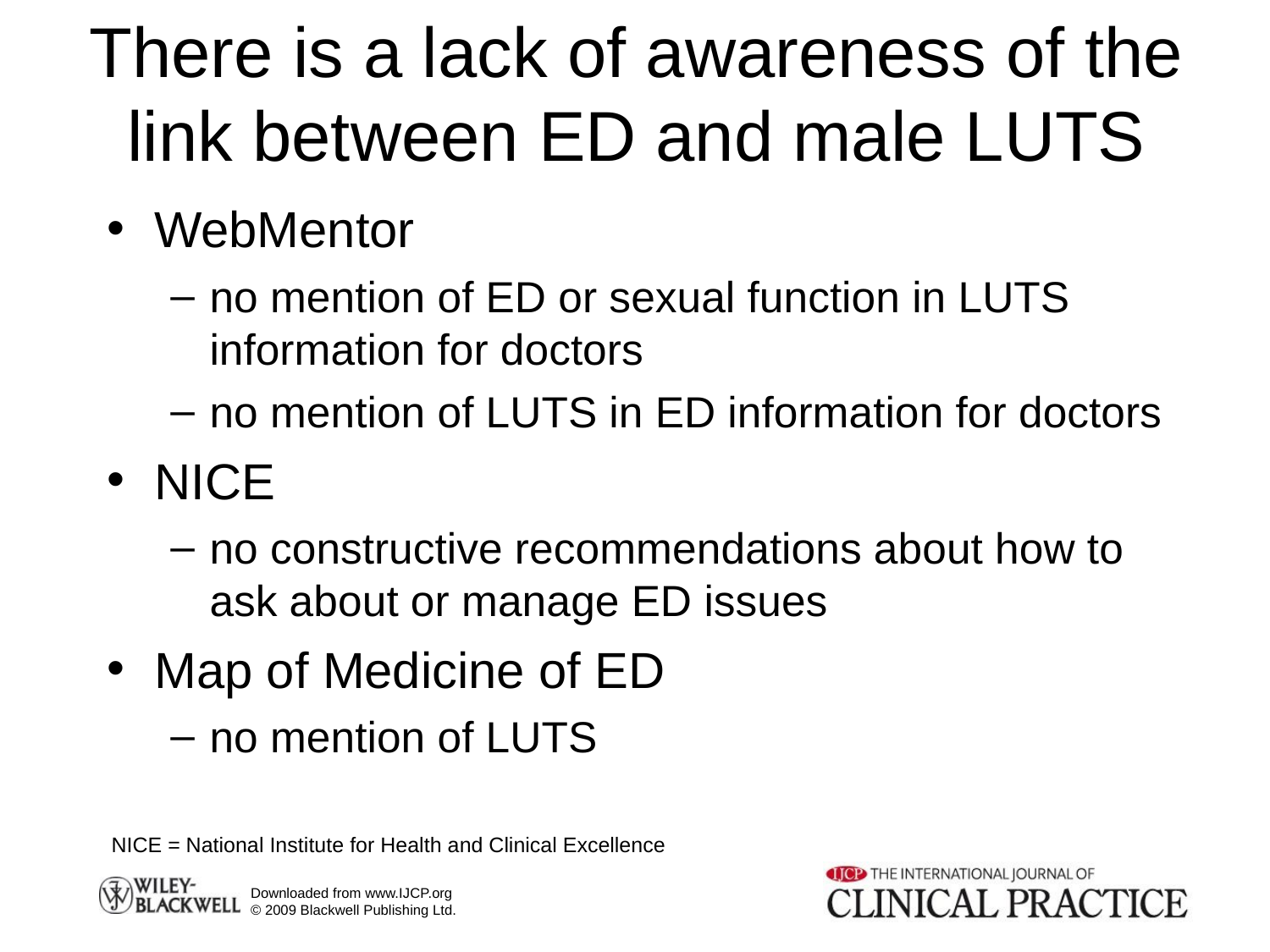

# There is a lack of awareness of the link between ED and male LUTS
WebMentor
no mention of ED or sexual function in LUTS information for doctors
no mention of LUTS in ED information for doctors
NICE
no constructive recommendations about how to ask about or manage ED issues
Map of Medicine of ED
no mention of LUTS
NICE = National Institute for Health and Clinical Excellence

## Slide 8
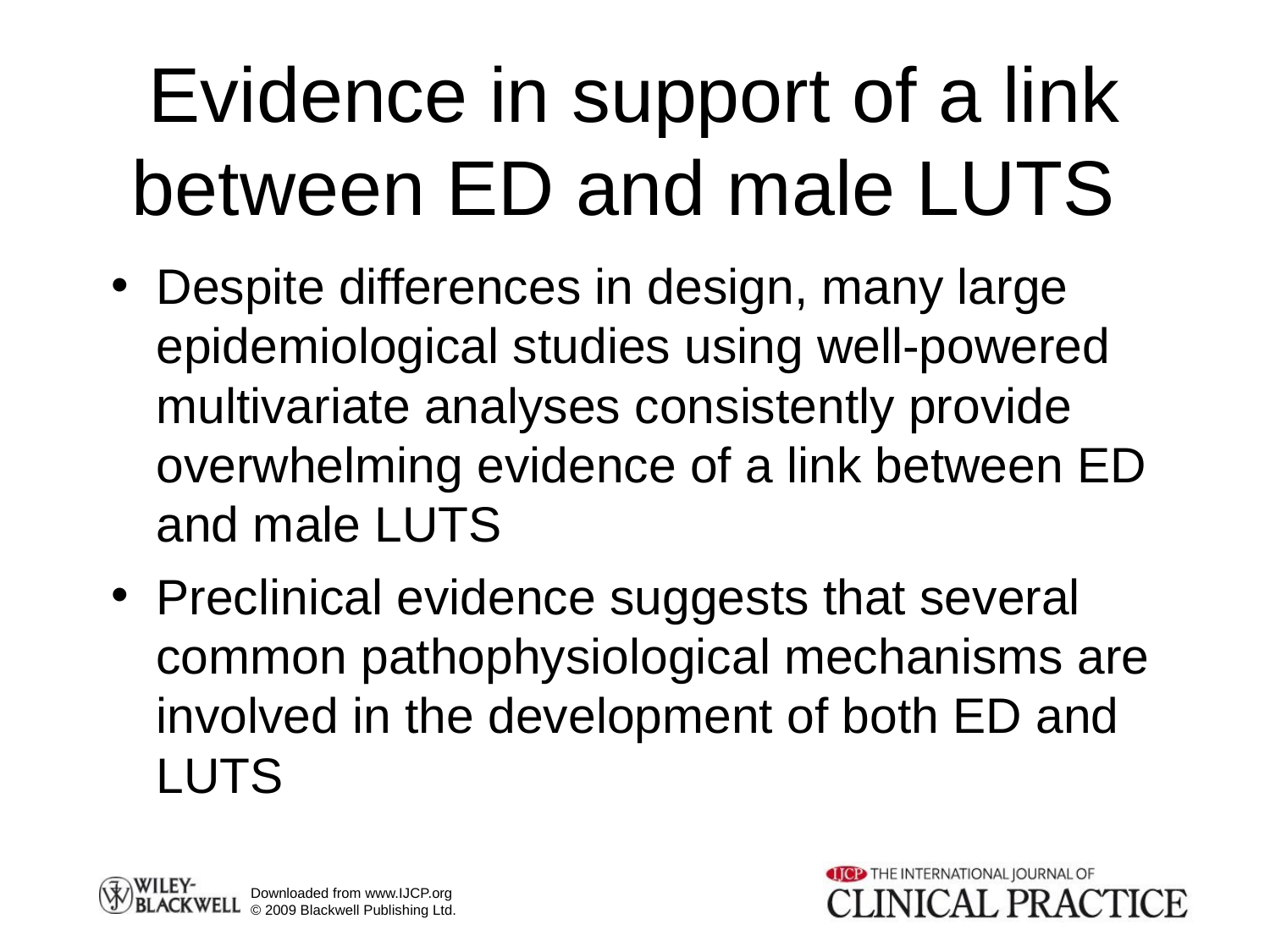

# Evidence in support of a link between ED and male LUTS
Despite differences in design, many large epidemiological studies using well-powered multivariate analyses consistently provide overwhelming evidence of a link between ED and male LUTS
Preclinical evidence suggests that several common pathophysiological mechanisms are involved in the development of both ED and LUTS

## Slide 9
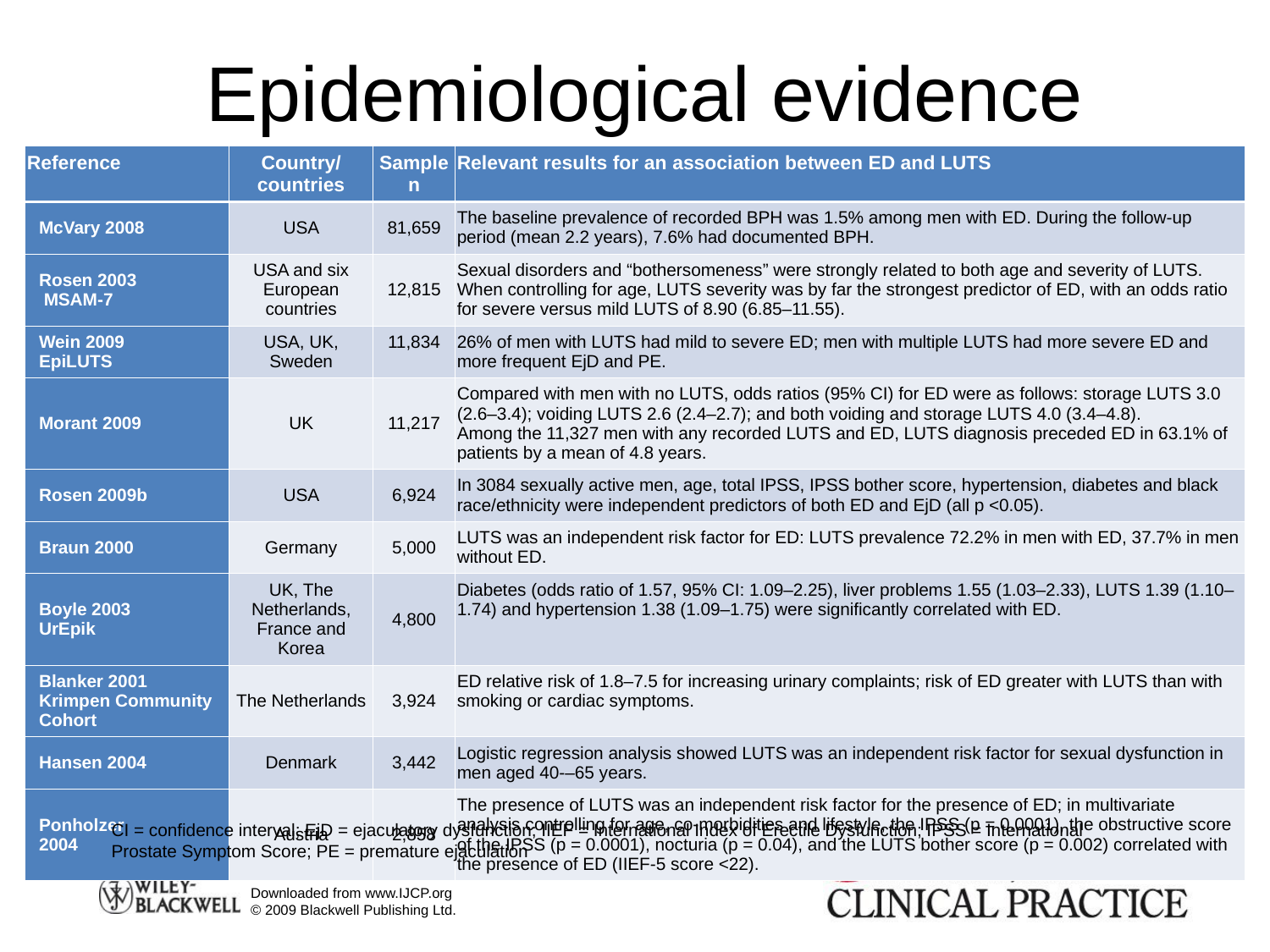

# Epidemiological evidence
| Reference | Country/ countries | Sample n | Relevant results for an association between ED and LUTS |
| --- | --- | --- | --- |
| McVary 2008 | USA | 81,659 | The baseline prevalence of recorded BPH was 1.5% among men with ED. During the follow-up period (mean 2.2 years), 7.6% had documented BPH. |
| Rosen 2003  MSAM-7 | USA and six European countries | 12,815 | Sexual disorders and “bothersomeness” were strongly related to both age and severity of LUTS. When controlling for age, LUTS severity was by far the strongest predictor of ED, with an odds ratio for severe versus mild LUTS of 8.90 (6.85–11.55). |
| Wein 2009 EpiLUTS | USA, UK, Sweden | 11,834 | 26% of men with LUTS had mild to severe ED; men with multiple LUTS had more severe ED and more frequent EjD and PE. |
| Morant 2009 | UK | 11,217 | Compared with men with no LUTS, odds ratios (95% CI) for ED were as follows: storage LUTS 3.0 (2.6–3.4); voiding LUTS 2.6 (2.4–2.7); and both voiding and storage LUTS 4.0 (3.4–4.8). Among the 11,327 men with any recorded LUTS and ED, LUTS diagnosis preceded ED in 63.1% of patients by a mean of 4.8 years. |
| Rosen 2009b | USA | 6,924 | In 3084 sexually active men, age, total IPSS, IPSS bother score, hypertension, diabetes and black race/ethnicity were independent predictors of both ED and EjD (all p <0.05). |
| Braun 2000 | Germany | 5,000 | LUTS was an independent risk factor for ED: LUTS prevalence 72.2% in men with ED, 37.7% in men without ED. |
| Boyle 2003 UrEpik | UK, The Netherlands, France and Korea | 4,800 | Diabetes (odds ratio of 1.57, 95% CI: 1.09–2.25), liver problems 1.55 (1.03–2.33), LUTS 1.39 (1.10–1.74) and hypertension 1.38 (1.09–1.75) were significantly correlated with ED. |
| Blanker 2001 Krimpen Community Cohort | The Netherlands | 3,924 | ED relative risk of 1.8–7.5 for increasing urinary complaints; risk of ED greater with LUTS than with smoking or cardiac symptoms. |
| Hansen 2004 | Denmark | 3,442 | Logistic regression analysis showed LUTS was an independent risk factor for sexual dysfunction in men aged 40-–65 years. |
| Ponholzer 2004 | Austria | 2,858 | The presence of LUTS was an independent risk factor for the presence of ED; in multivariate analysis controlling for age, co-morbidities and lifestyle, the IPSS (p = 0.0001), the obstructive score of the IPSS (p = 0.0001), nocturia (p = 0.04), and the LUTS bother score (p = 0.002) correlated with the presence of ED (IIEF-5 score <22). |
CI = confidence interval; EjD = ejaculatory dysfunction; IIEF = International Index of Erectile Dysfunction; IPSS = International Prostate Symptom Score; PE = premature ejaculation

## Slide 10
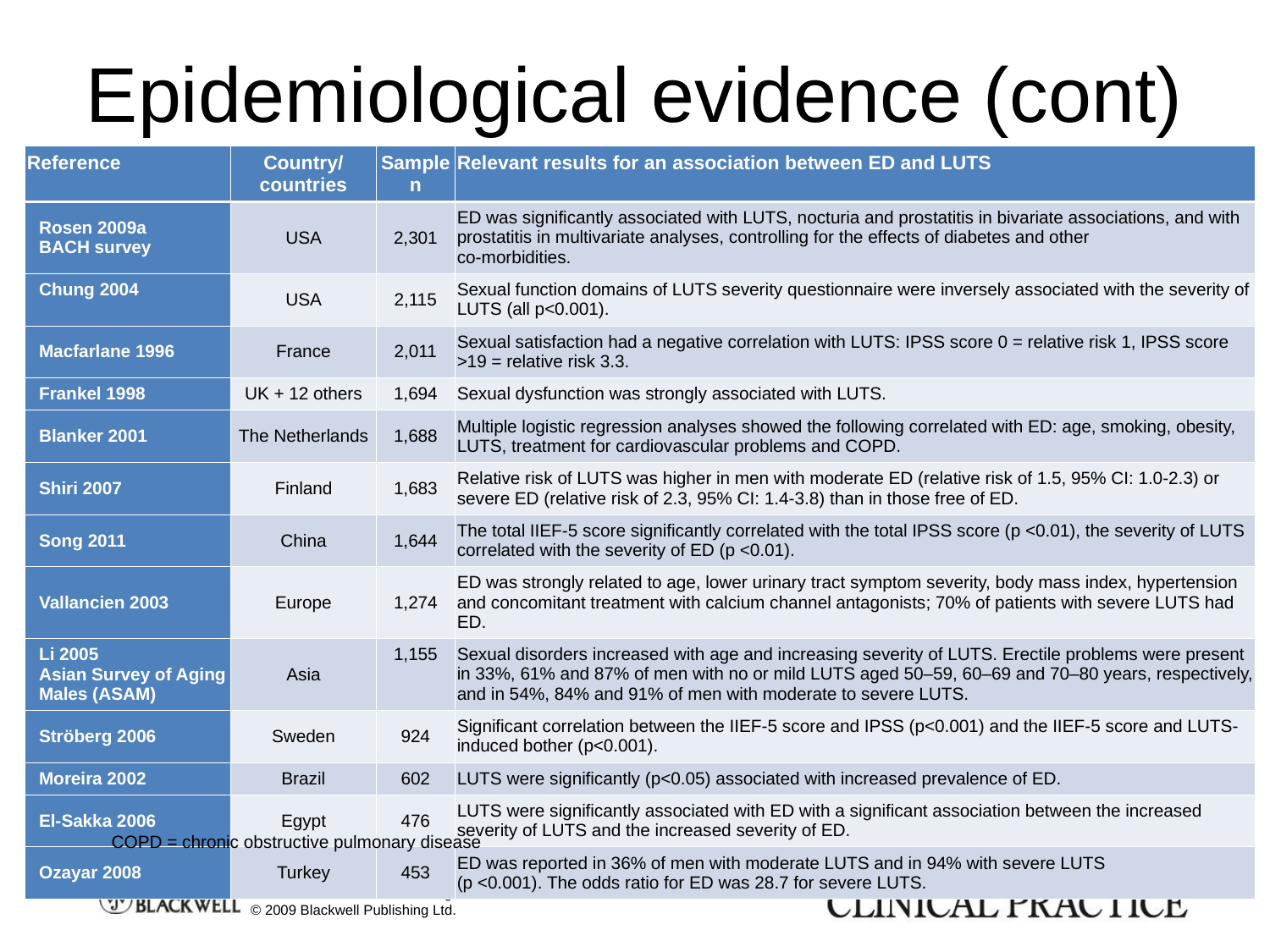

# Epidemiological evidence (cont)
| Reference | Country/ countries | Sample n | Relevant results for an association between ED and LUTS |
| --- | --- | --- | --- |
| Rosen 2009a BACH survey | USA | 2,301 | ED was significantly associated with LUTS, nocturia and prostatitis in bivariate associations, and with prostatitis in multivariate analyses, controlling for the effects of diabetes and other co-morbidities. |
| Chung 2004 | USA | 2,115 | Sexual function domains of LUTS severity questionnaire were inversely associated with the severity of LUTS (all p<0.001). |
| Macfarlane 1996 | France | 2,011 | Sexual satisfaction had a negative correlation with LUTS: IPSS score 0 = relative risk 1, IPSS score >19 = relative risk 3.3. |
| Frankel 1998 | UK + 12 others | 1,694 | Sexual dysfunction was strongly associated with LUTS. |
| Blanker 2001 | The Netherlands | 1,688 | Multiple logistic regression analyses showed the following correlated with ED: age, smoking, obesity, LUTS, treatment for cardiovascular problems and COPD. |
| Shiri 2007 | Finland | 1,683 | Relative risk of LUTS was higher in men with moderate ED (relative risk of 1.5, 95% CI: 1.0-2.3) or severe ED (relative risk of 2.3, 95% CI: 1.4-3.8) than in those free of ED. |
| Song 2011 | China | 1,644 | The total IIEF-5 score significantly correlated with the total IPSS score (p <0.01), the severity of LUTS correlated with the severity of ED (p <0.01). |
| Vallancien 2003 | Europe | 1,274 | ED was strongly related to age, lower urinary tract symptom severity, body mass index, hypertension and concomitant treatment with calcium channel antagonists; 70% of patients with severe LUTS had ED. |
| Li 2005 Asian Survey of Aging Males (ASAM) | Asia | 1,155 | Sexual disorders increased with age and increasing severity of LUTS. Erectile problems were present in 33%, 61% and 87% of men with no or mild LUTS aged 50–59, 60–69 and 70–80 years, respectively, and in 54%, 84% and 91% of men with moderate to severe LUTS. |
| Ströberg 2006 | Sweden | 924 | Significant correlation between the IIEF-5 score and IPSS (p<0.001) and the IIEF-5 score and LUTS-induced bother (p<0.001). |
| Moreira 2002 | Brazil | 602 | LUTS were significantly (p<0.05) associated with increased prevalence of ED. |
| El-Sakka 2006 | Egypt | 476 | LUTS were significantly associated with ED with a significant association between the increased severity of LUTS and the increased severity of ED. |
| Ozayar 2008 | Turkey | 453 | ED was reported in 36% of men with moderate LUTS and in 94% with severe LUTS (p <0.001). The odds ratio for ED was 28.7 for severe LUTS. |
COPD = chronic obstructive pulmonary disease

## Slide 11
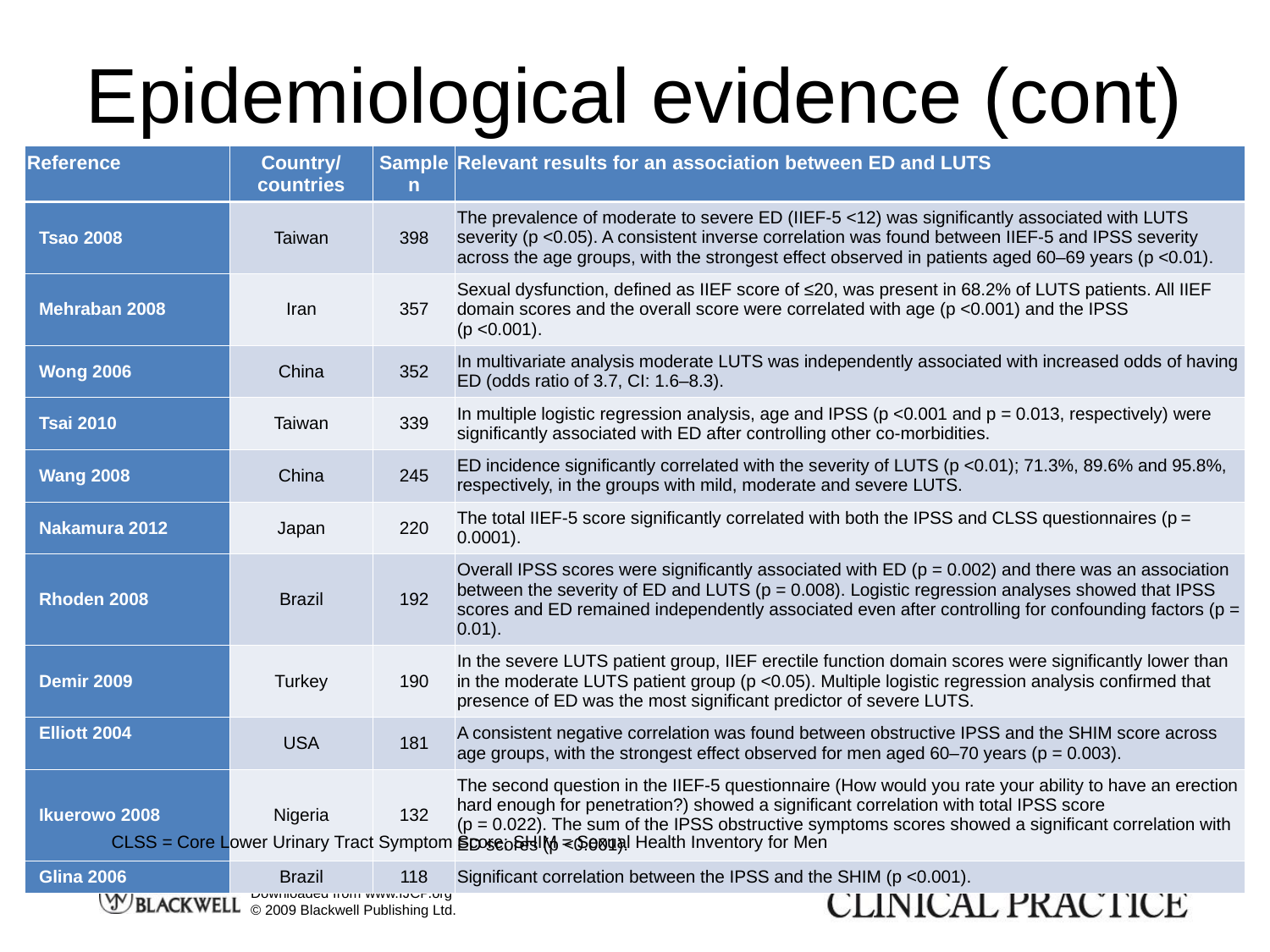

# Epidemiological evidence (cont)
| Reference | Country/ countries | Sample n | Relevant results for an association between ED and LUTS |
| --- | --- | --- | --- |
| Tsao 2008 | Taiwan | 398 | The prevalence of moderate to severe ED (IIEF-5 <12) was significantly associated with LUTS severity (p <0.05). A consistent inverse correlation was found between IIEF-5 and IPSS severity across the age groups, with the strongest effect observed in patients aged 60–69 years (p <0.01). |
| Mehraban 2008 | Iran | 357 | Sexual dysfunction, defined as IIEF score of ≤20, was present in 68.2% of LUTS patients. All IIEF domain scores and the overall score were correlated with age (p <0.001) and the IPSS (p <0.001). |
| Wong 2006 | China | 352 | In multivariate analysis moderate LUTS was independently associated with increased odds of having ED (odds ratio of 3.7, CI: 1.6–8.3). |
| Tsai 2010 | Taiwan | 339 | In multiple logistic regression analysis, age and IPSS (p <0.001 and p = 0.013, respectively) were significantly associated with ED after controlling other co-morbidities. |
| Wang 2008 | China | 245 | ED incidence significantly correlated with the severity of LUTS (p <0.01); 71.3%, 89.6% and 95.8%, respectively, in the groups with mild, moderate and severe LUTS. |
| Nakamura 2012 | Japan | 220 | The total IIEF-5 score significantly correlated with both the IPSS and CLSS questionnaires (p = 0.0001). |
| Rhoden 2008 | Brazil | 192 | Overall IPSS scores were significantly associated with ED (p = 0.002) and there was an association between the severity of ED and LUTS (p = 0.008). Logistic regression analyses showed that IPSS scores and ED remained independently associated even after controlling for confounding factors (p = 0.01). |
| Demir 2009 | Turkey | 190 | In the severe LUTS patient group, IIEF erectile function domain scores were significantly lower than in the moderate LUTS patient group (p <0.05). Multiple logistic regression analysis confirmed that presence of ED was the most significant predictor of severe LUTS. |
| Elliott 2004 | USA | 181 | A consistent negative correlation was found between obstructive IPSS and the SHIM score across age groups, with the strongest effect observed for men aged 60–70 years (p = 0.003). |
| Ikuerowo 2008 | Nigeria | 132 | The second question in the IIEF-5 questionnaire (How would you rate your ability to have an erection hard enough for penetration?) showed a significant correlation with total IPSS score (p = 0.022). The sum of the IPSS obstructive symptoms scores showed a significant correlation with ED scores (p <0.001). |
| Glina 2006 | Brazil | 118 | Significant correlation between the IPSS and the SHIM (p <0.001). |
CLSS = Core Lower Urinary Tract Symptom Score; SHIM = Sexual Health Inventory for Men

## Slide 12
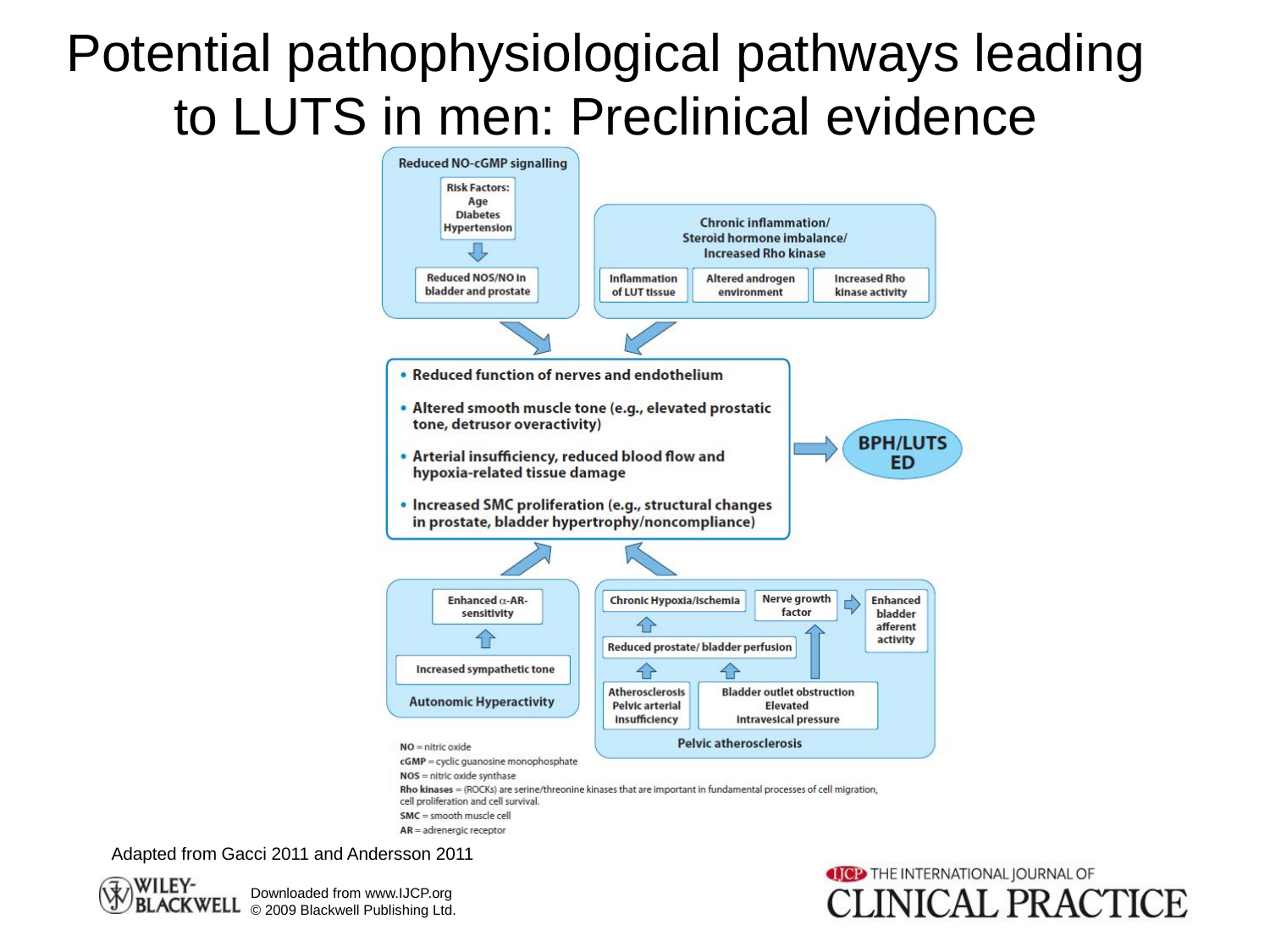

# Potential pathophysiological pathways leading to LUTS in men: Preclinical evidence
Adapted from Gacci 2011 and Andersson 2011

## Slide 13
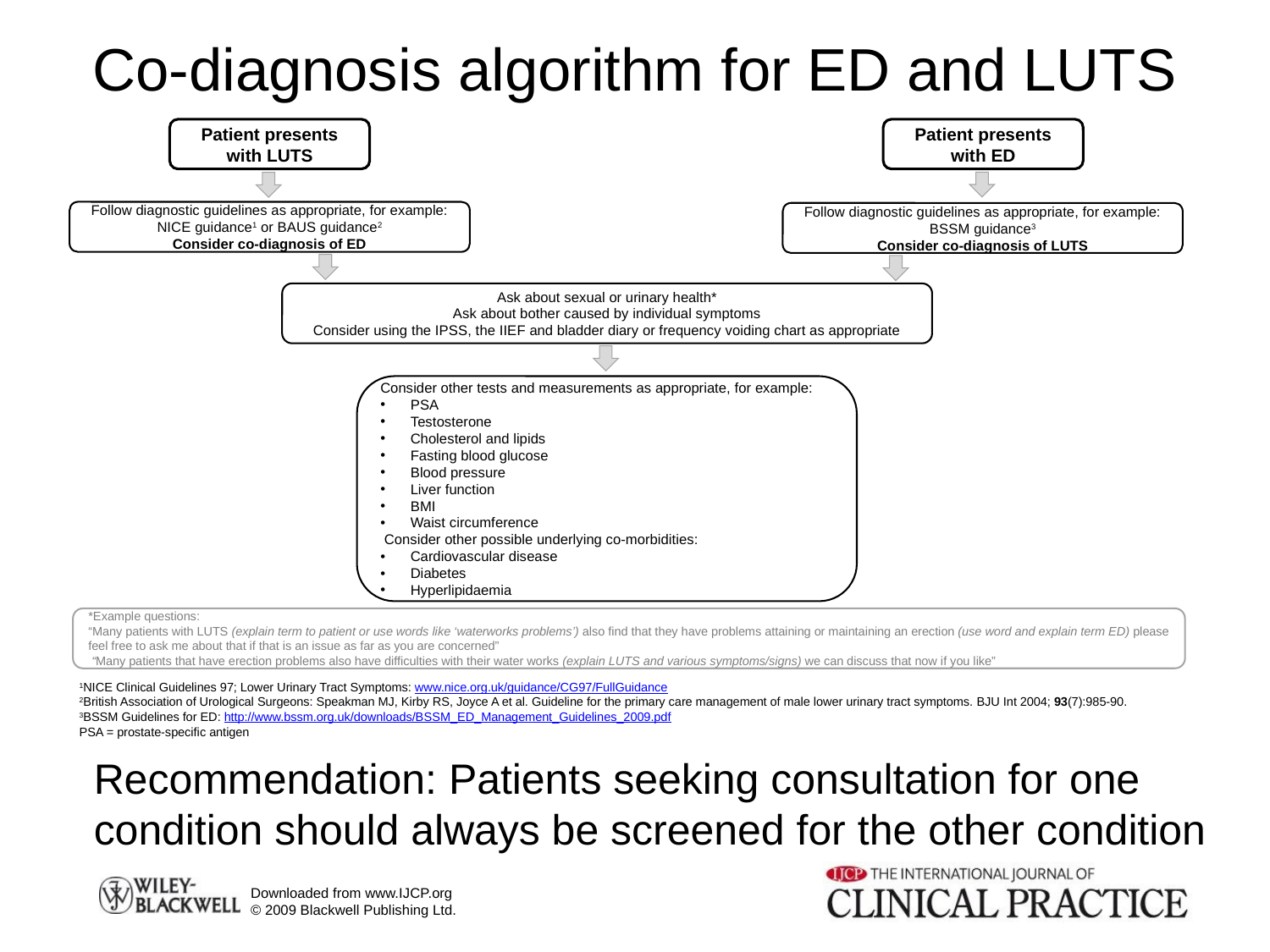

# Co-diagnosis algorithm for ED and LUTS
Patient presents with LUTS
Patient presents with ED
Follow diagnostic guidelines as appropriate, for example:
NICE guidance1 or BAUS guidance2
Consider co-diagnosis of ED
Follow diagnostic guidelines as appropriate, for example:
BSSM guidance3
Consider co-diagnosis of LUTS
Ask about sexual or urinary health*
Ask about bother caused by individual symptoms
Consider using the IPSS, the IIEF and bladder diary or frequency voiding chart as appropriate
Consider other tests and measurements as appropriate, for example:
PSA
Testosterone
Cholesterol and lipids
Fasting blood glucose
Blood pressure
Liver function
BMI
Waist circumference
 Consider other possible underlying co-morbidities:
Cardiovascular disease
Diabetes
Hyperlipidaemia
*Example questions:
“Many patients with LUTS (explain term to patient or use words like ‘waterworks problems’) also find that they have problems attaining or maintaining an erection (use word and explain term ED) please feel free to ask me about that if that is an issue as far as you are concerned”
 “Many patients that have erection problems also have difficulties with their water works (explain LUTS and various symptoms/signs) we can discuss that now if you like”
1NICE Clinical Guidelines 97; Lower Urinary Tract Symptoms: www.nice.org.uk/guidance/CG97/FullGuidance
2British Association of Urological Surgeons: Speakman MJ, Kirby RS, Joyce A et al. Guideline for the primary care management of male lower urinary tract symptoms. BJU Int 2004; 93(7):985-90.
3BSSM Guidelines for ED: http://www.bssm.org.uk/downloads/BSSM_ED_Management_Guidelines_2009.pdf
PSA = prostate-specific antigen
Recommendation: Patients seeking consultation for one condition should always be screened for the other condition

## Slide 14
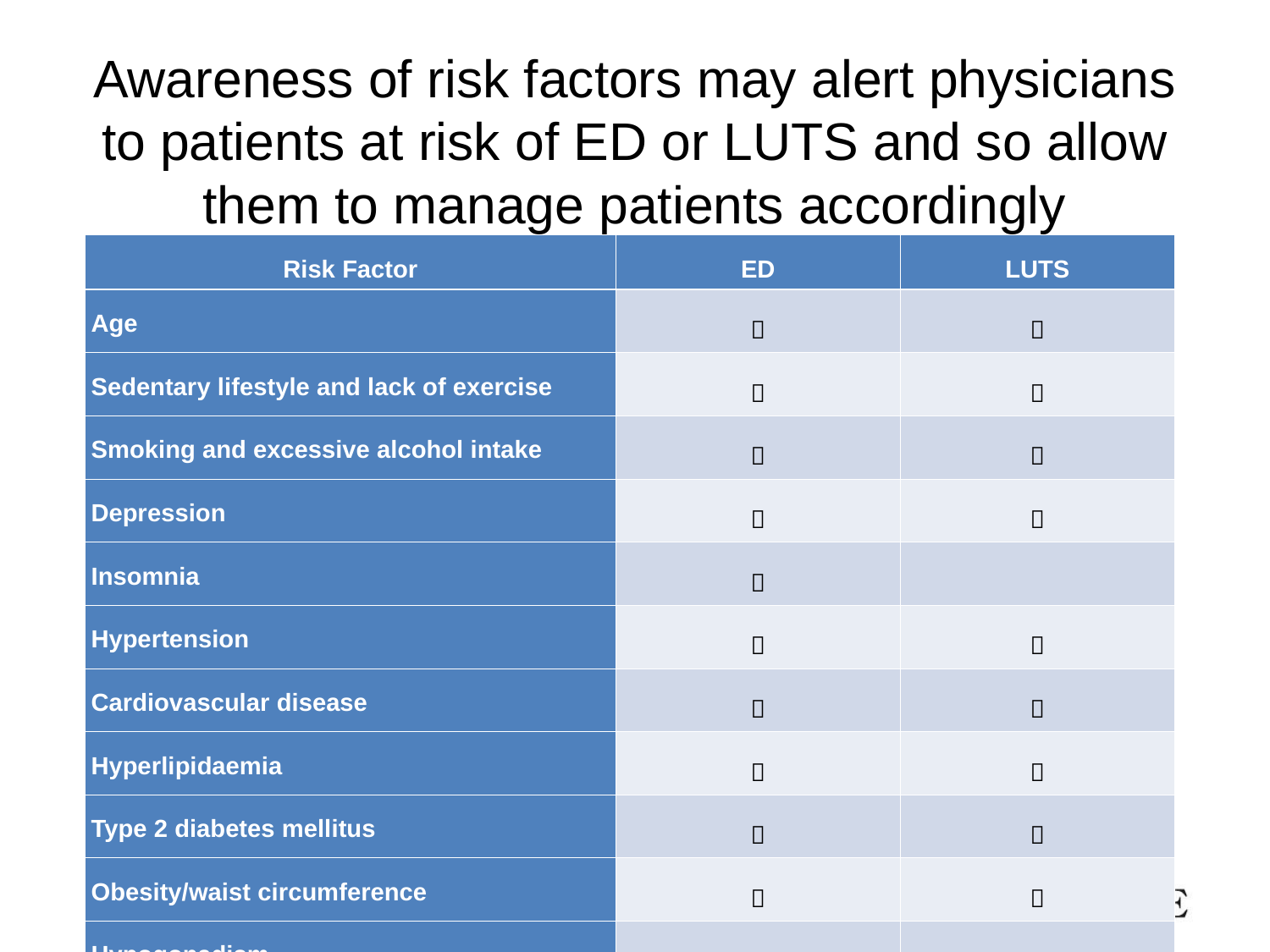

# Awareness of risk factors may alert physicians to patients at risk of ED or LUTS and so allow them to manage patients accordingly
| Risk Factor | ED | LUTS |
| --- | --- | --- |
| Age |  |  |
| Sedentary lifestyle and lack of exercise |  |  |
| Smoking and excessive alcohol intake |  |  |
| Depression |  |  |
| Insomnia |  | |
| Hypertension |  |  |
| Cardiovascular disease |  |  |
| Hyperlipidaemia |  |  |
| Type 2 diabetes mellitus |  |  |
| Obesity/waist circumference |  |  |
| Hypogonadism |  |  |
| Prostate disorder |  |  |
| Inflammation | |  |

## Slide 15
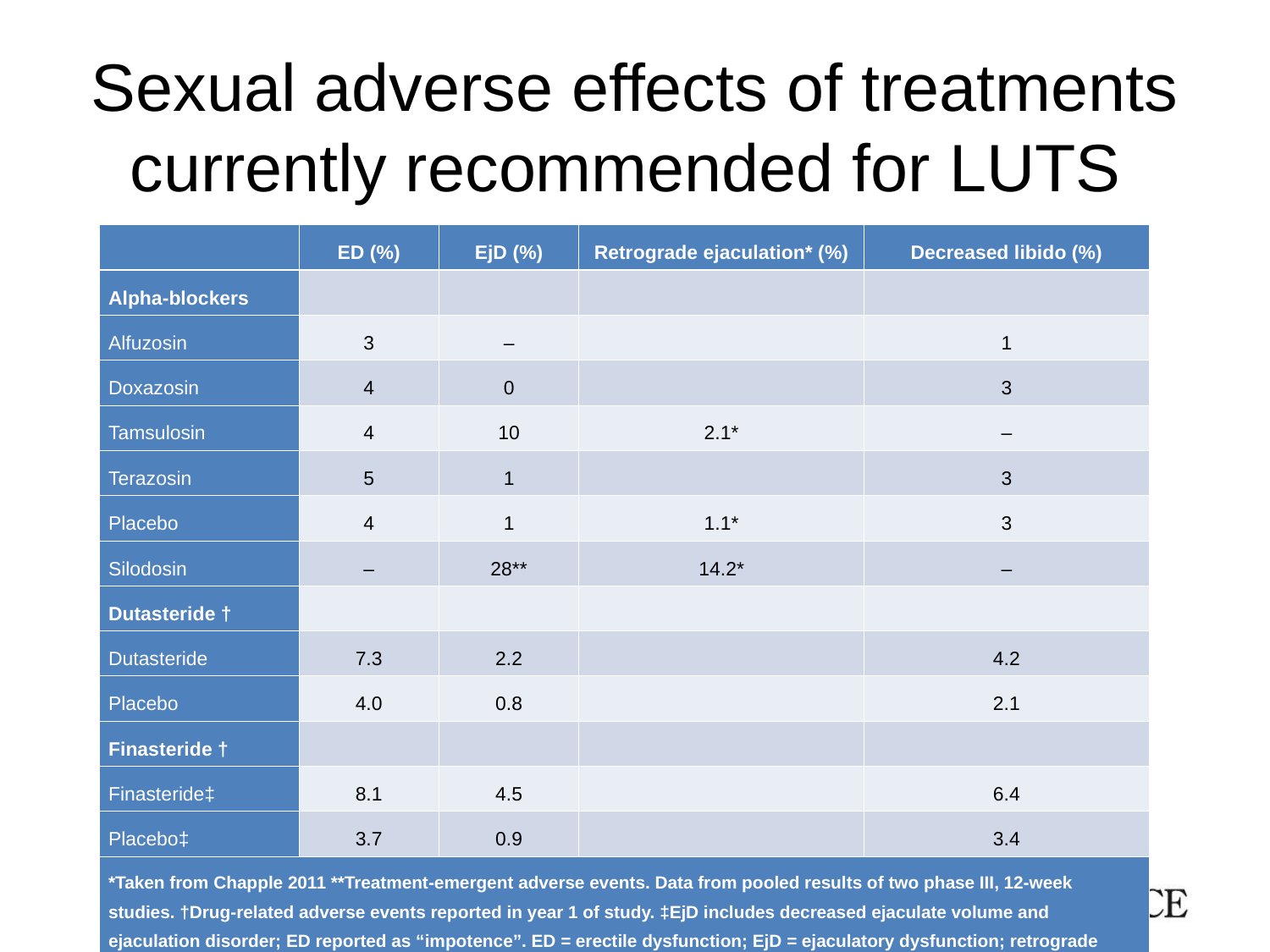

# Sexual adverse effects of treatments currently recommended for LUTS
| | ED (%) | EjD (%) | Retrograde ejaculation\* (%) | Decreased libido (%) |
| --- | --- | --- | --- | --- |
| Alpha-blockers | | | | |
| Alfuzosin | 3 | – | | 1 |
| Doxazosin | 4 | 0 | | 3 |
| Tamsulosin | 4 | 10 | 2.1\* | – |
| Terazosin | 5 | 1 | | 3 |
| Placebo | 4 | 1 | 1.1\* | 3 |
| Silodosin | – | 28\*\* | 14.2\* | – |
| Dutasteride † | | | | |
| Dutasteride | 7.3 | 2.2 | | 4.2 |
| Placebo | 4.0 | 0.8 | | 2.1 |
| Finasteride † | | | | |
| Finasteride‡ | 8.1 | 4.5 | | 6.4 |
| Placebo‡ | 3.7 | 0.9 | | 3.4 |
| \*Taken from Chapple 2011 \*\*Treatment-emergent adverse events. Data from pooled results of two phase III, 12-week studies. †Drug-related adverse events reported in year 1 of study. ‡EjD includes decreased ejaculate volume and ejaculation disorder; ED reported as “impotence”. ED = erectile dysfunction; EjD = ejaculatory dysfunction; retrograde ejaculation=orgasm with no semen, orgasm semen quantity reduced, and retrograde ejaculation | | | | |

## Slide 16
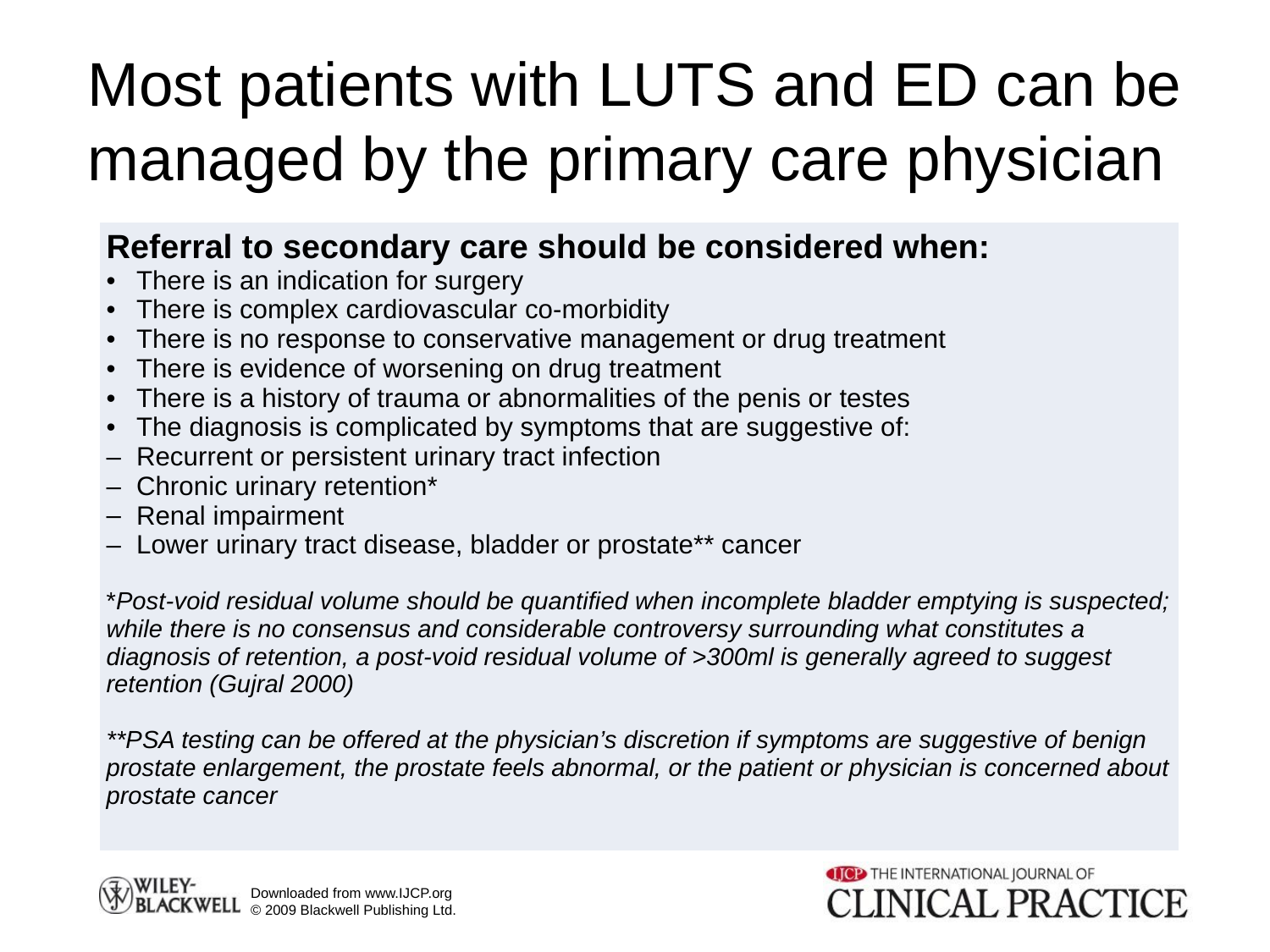

# Most patients with LUTS and ED can be managed by the primary care physician
| Referral to secondary care should be considered when: There is an indication for surgery There is complex cardiovascular co-morbidity There is no response to conservative management or drug treatment There is evidence of worsening on drug treatment There is a history of trauma or abnormalities of the penis or testes The diagnosis is complicated by symptoms that are suggestive of: Recurrent or persistent urinary tract infection Chronic urinary retention\* Renal impairment Lower urinary tract disease, bladder or prostate\*\* cancer \*Post-void residual volume should be quantified when incomplete bladder emptying is suspected; while there is no consensus and considerable controversy surrounding what constitutes a diagnosis of retention, a post-void residual volume of >300ml is generally agreed to suggest retention (Gujral 2000) \*\*PSA testing can be offered at the physician’s discretion if symptoms are suggestive of benign prostate enlargement, the prostate feels abnormal, or the patient or physician is concerned about prostate cancer |
| --- |

## Slide 17
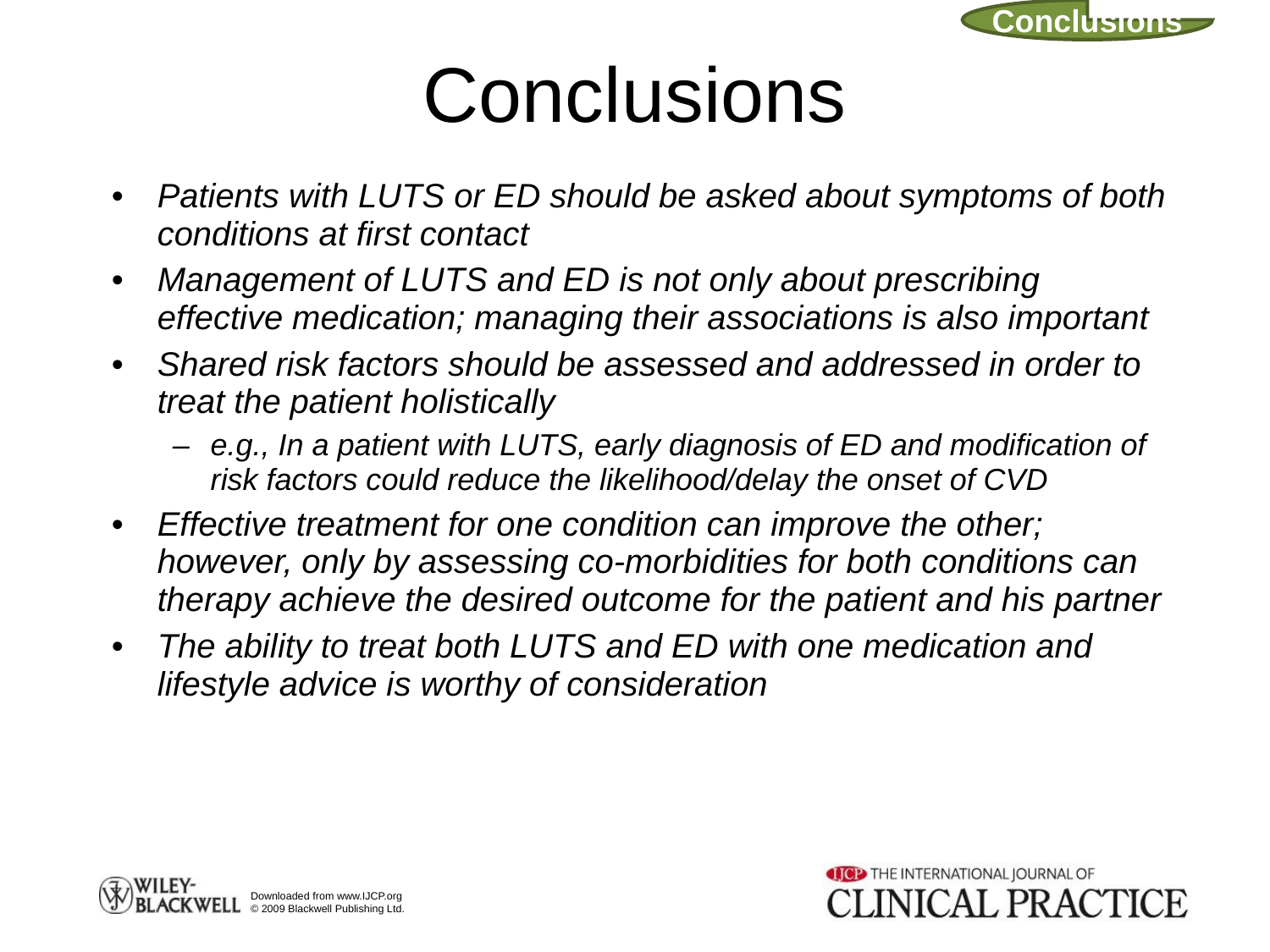

# Conclusions
Patients with LUTS or ED should be asked about symptoms of both conditions at first contact
Management of LUTS and ED is not only about prescribing effective medication; managing their associations is also important
Shared risk factors should be assessed and addressed in order to treat the patient holistically
e.g., In a patient with LUTS, early diagnosis of ED and modification of risk factors could reduce the likelihood/delay the onset of CVD
Effective treatment for one condition can improve the other; however, only by assessing co-morbidities for both conditions can therapy achieve the desired outcome for the patient and his partner
The ability to treat both LUTS and ED with one medication and lifestyle advice is worthy of consideration

## Slide 18
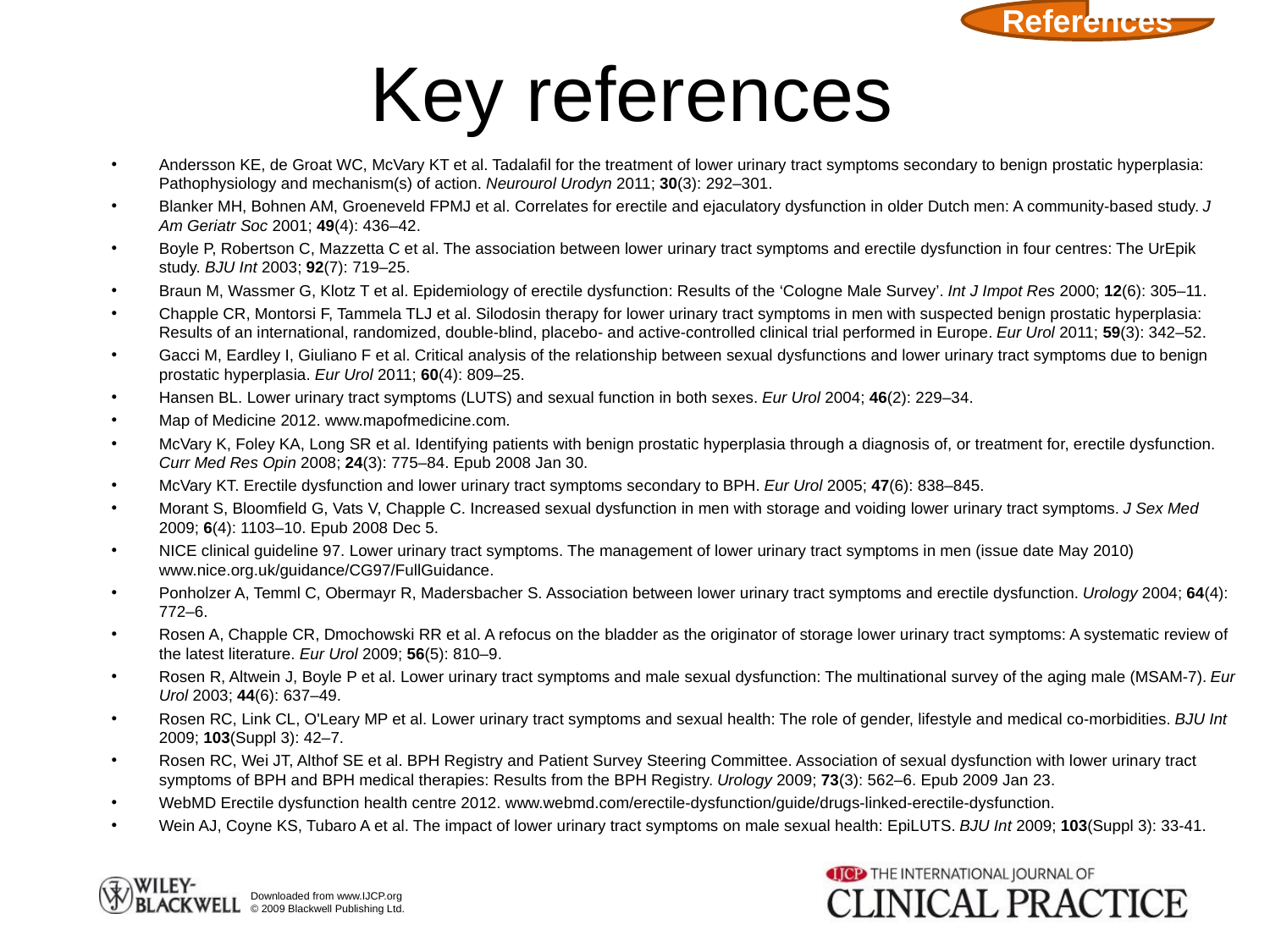

# Key references
Andersson KE, de Groat WC, McVary KT et al. Tadalafil for the treatment of lower urinary tract symptoms secondary to benign prostatic hyperplasia: Pathophysiology and mechanism(s) of action. Neurourol Urodyn 2011; 30(3): 292–301.
Blanker MH, Bohnen AM, Groeneveld FPMJ et al. Correlates for erectile and ejaculatory dysfunction in older Dutch men: A community-based study. J Am Geriatr Soc 2001; 49(4): 436–42.
Boyle P, Robertson C, Mazzetta C et al. The association between lower urinary tract symptoms and erectile dysfunction in four centres: The UrEpik study. BJU Int 2003; 92(7): 719–25.
Braun M, Wassmer G, Klotz T et al. Epidemiology of erectile dysfunction: Results of the ‘Cologne Male Survey’. Int J Impot Res 2000; 12(6): 305–11.
Chapple CR, Montorsi F, Tammela TLJ et al. Silodosin therapy for lower urinary tract symptoms in men with suspected benign prostatic hyperplasia: Results of an international, randomized, double-blind, placebo- and active-controlled clinical trial performed in Europe. Eur Urol 2011; 59(3): 342–52.
Gacci M, Eardley I, Giuliano F et al. Critical analysis of the relationship between sexual dysfunctions and lower urinary tract symptoms due to benign prostatic hyperplasia. Eur Urol 2011; 60(4): 809–25.
Hansen BL. Lower urinary tract symptoms (LUTS) and sexual function in both sexes. Eur Urol 2004; 46(2): 229–34.
Map of Medicine 2012. www.mapofmedicine.com.
McVary K, Foley KA, Long SR et al. Identifying patients with benign prostatic hyperplasia through a diagnosis of, or treatment for, erectile dysfunction. Curr Med Res Opin 2008; 24(3): 775–84. Epub 2008 Jan 30.
McVary KT. Erectile dysfunction and lower urinary tract symptoms secondary to BPH. Eur Urol 2005; 47(6): 838–845.
Morant S, Bloomfield G, Vats V, Chapple C. Increased sexual dysfunction in men with storage and voiding lower urinary tract symptoms. J Sex Med 2009; 6(4): 1103–10. Epub 2008 Dec 5.
NICE clinical guideline 97. Lower urinary tract symptoms. The management of lower urinary tract symptoms in men (issue date May 2010) www.nice.org.uk/guidance/CG97/FullGuidance.
Ponholzer A, Temml C, Obermayr R, Madersbacher S. Association between lower urinary tract symptoms and erectile dysfunction. Urology 2004; 64(4): 772–6.
Rosen A, Chapple CR, Dmochowski RR et al. A refocus on the bladder as the originator of storage lower urinary tract symptoms: A systematic review of the latest literature. Eur Urol 2009; 56(5): 810–9.
Rosen R, Altwein J, Boyle P et al. Lower urinary tract symptoms and male sexual dysfunction: The multinational survey of the aging male (MSAM-7). Eur Urol 2003; 44(6): 637–49.
Rosen RC, Link CL, O'Leary MP et al. Lower urinary tract symptoms and sexual health: The role of gender, lifestyle and medical co-morbidities. BJU Int 2009; 103(Suppl 3): 42–7.
Rosen RC, Wei JT, Althof SE et al. BPH Registry and Patient Survey Steering Committee. Association of sexual dysfunction with lower urinary tract symptoms of BPH and BPH medical therapies: Results from the BPH Registry. Urology 2009; 73(3): 562–6. Epub 2009 Jan 23.
WebMD Erectile dysfunction health centre 2012. www.webmd.com/erectile-dysfunction/guide/drugs-linked-erectile-dysfunction.
Wein AJ, Coyne KS, Tubaro A et al. The impact of lower urinary tract symptoms on male sexual health: EpiLUTS. BJU Int 2009; 103(Suppl 3): 33-41.
